# Network targeting combination therapy of synthetic lethal vulnerabilities in *9p21-*deficient glioblastoma: A case report

**DOI:** 10.1093/noajnl/vdad162

**Published:** 2023-12-10

**Authors:** Michael P Castro, Kristin Dittmar

**Affiliations:** Department of Oncology, Personalized Cancer Medicine, PLLC, Los Angeles, California, USA; Department of Oncology, Beverly Hills Cancer Center, Beverly Hills, California, USA; Cellworks Group, Inc., San Francisco, California, USA; Department of Radiology, Beverly Hills Cancer Center, Beverly Hills, California, USA

**Keywords:** exceptional responders, glioblastoma, network targeting combination therapy (NTCT), synthetic lethality

## Abstract

**Background:**

Patients with relapsed or progressive glioblastoma only rarely respond to salvage therapies. Nevertheless, comprehensive genomic profiling can provide insight that can identify promising approaches. Signaling pathway analyses have revealed synthetic lethal partnerships, which create the possibility of targeting vulnerabilities arising from the loss of tumor suppressor genes. For synthetic lethal vulnerabilities that are not present in normal tissues, lethal cytotoxicity against cancer cells can be achieved without the necessity of causing normal tissue toxicity. This case report describes a patient with progressive glioblastoma with homozygous deletion of chromosome *9p21*.

**Methods and Results:**

Vulnerabilities created by *CDKN2A* and *MTAP* loss were exploited with pemetrexed, bevacizumab, and candesartan to achieve a clinically meaningful remission by targeting multiple synthetic lethal nodes.

**Conclusion:**

Synthetic lethality can reveal the basis for exceptional responsiveness, thus extending the utility of molecular profiling and fulfilling the promise of precision medicine.

## Background

Despite enormous efforts, therapy for glioblastoma (GBM) remains inadequate. For more than half of patients with O6–methylguanine methyltransferase (*MGMT*) unmethylated cancers, fewer than 15% derive benefit from temozolomide (TMZ).^1^ Among *MGMT* methylated patients, as many as half may have progressive disease while receiving the standard of care.^2^ In the relapsed or progressive disease setting, no routinely effective therapy has emerged. A tiny minority of GBM may have oncogene drivers that can be targeted with tyrosine kinase inhibitors. More commonly, these cancers have complex proteogenomic networks that defy single gene–single drug targeting strategies, but may possess disease-specific synthetic lethal (SL) treatment nodes, that is, Achilles’ heel vulnerabilities, that can be exploited in therapy design.

**Figure 1.**
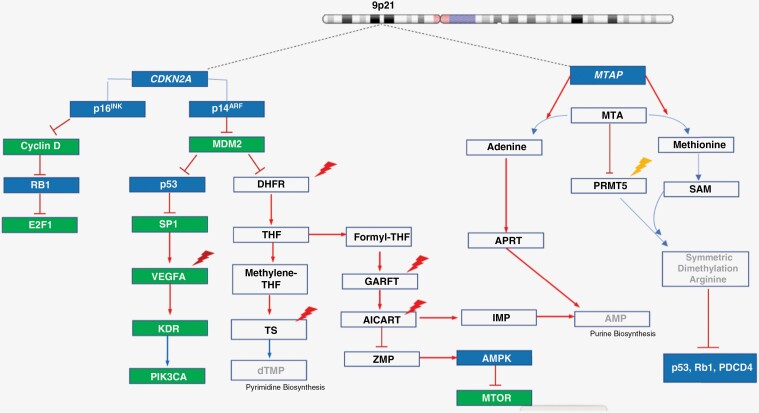
*9p21* deletion signaling network representing loss of *CDKN2A* and *MTAP*. *CDKN2A* codes for 2 tumor suppressors, p14^ARF^ and p16^INK^. p14^ARF^ sequesters MDM2 in the nucleolus. MDM2 also targets DHFR, inhibiting its enzymatic activity through monoubiquitination.^4^ DHFR catalyzes the formation of THF, a precursor of methylene-THF, the necessary cofactor for dTMP in pyrimidine biosynthesis and formyl-THF, a cofactor of GARFT in the formation of IMP and AMP in purine biosynthesis. Pemetrexed also targets AICART which results in accumulation of ZMP, an activator of AMPK, a key regulator of MTOR signaling.^5^ Enhanced MDM2-mediated ubiquitin turnover of p53 from loss of p14^ARF^ removes p53 as a brake on SP1-mediated *VEGFA* transcription.^6^ Loss of *MTAP* results in the accumulation of methylthioadenosine (MTA). Both MTAP and GARFT are responsible for AMP synthesis, such that *MTAP* deletion creates exquisite sensitivity to adenosine depletion from GARFT targeting.^7^ PRMT5 is also a SL partner of *MTAP.*^8^ Elevated MTA arising from *MTAP* loss inhibits PRMT5 by competitively binding to the SAM cofactor binding site, thus compromising symmetric dimethylation reactions that support malignancy. Tumor suppressors shown in blue and oncogenes shown in green. Thunderbolts show sites of action of bevacizumab (red), pemetrexed (orange), and candesartan (yellow).

Though biomarkers for cytotoxic agents have been challenging to develop, recent insights predict unusual sensitivity to cytotoxic agents based on synthetic lethality. Chromosome *9p21* is deleted in approximately 40%–50% of GBM as well as in 15%–20% of all human cancers. It contains the loci of 2 adjacent tumor suppressor genes, cyclin-dependent kinase inhibitor 2A (*CDKN2A*) and methylthioadenosine phosphorylase (*MTAP*), which are co-deleted in approximately 80%–90%.^3^ Remarkably, both genes possess SL vulnerabilities (Figure 1). While pemetrexed use in glioblastoma is unexplored, *CDKN2A* and *MTAP* loss have emerged as important predictors of pemetrexed benefit in other cancers. In non–small cell lung cancer (NSCLC) cell lines, *CDKN2A*-deficient cancers have up to 100-fold lower IC50 to pemetrexed compared to *CDKN2A*-proficient cancers.^9^ Among patients with NSCLC, *MTAP* deficiency predicts superior responses to pemetrexed including those with brain metastases.^10^ In *MTAP*-deficient bladder cancer cell lines, the IC50 of pemetrexed is up to 40-fold lower compared to *MTAP*-proficient cells.^11^ A recent report identified dramatic remission from pemetrexed in patients with locally advanced and metastatic chordoma harboring *CDKN2A* loss, a historically untreatable malignancy.^12^

The absence of p14^ARF^ enhances pemetrexed efficacy, producing Achilles’ heel vulnerabilities through dihydrofolate reductase (DHFR), glycinamide ribonucleotide formyltransferase (GARFT), and amino-imidazole carboxamide ribonucleotide formyltransferase (AICART), all pemetrexed targets. Another insight about *CDKN2A* relates to bevacizumab. Defying the generalized conclusion that bevacizumab does not impact survival in GBM, Japanese investigators uncovered a survival benefit for bevacizumab in *MGMT*-unmethylated, *CDKN2A*-deficient GBM,^13^ plausibly by blocking autocrine stimulation through the vascular endothelial growth factor (VEGFA)–KDR–PI3K pathway.^14^ Mechanistically, *MTAP* is also synthetic lethal with GARFT. Finally, protein arginine methyltransferase 5 (PRMT5) has emerged as an SL partner of MTAP. PRMT5 inhibitors have entered clinical trials to exploit this weakness. However, the angiotensin receptor blocker (ARB) candesartan has been reported to inhibit PRMT5 and precipitate cell death in *MTAP*-deficient cell lines.^15^ Whether candesartan produces an antitumor effect in the clinic remains to be demonstrated. Nevertheless, candesartan is useful for managing the side effects of hypertension caused by bevacizumab, and even a modest impact against PRMT5 could be of value in targeting *MTAP*-deficient cancers.

Relapsed GBM patients seldom achieve responses that satisfy Response Assessment in Neuro-oncology (RANO) criteria. Here we discuss the experience of using a combination of pemetrexed, bevacizumab, and candesartan (PBC) to attack multiple synthetic vulnerabilities created by homozygous *9p21* deletion in a GBM patient with refractory disease and a large tumor burden.

## Case Report

A 54-year-old, right-handed man presented with 6 months of progressive right hemiparesis and an MRI showing a large, heterogeneously enhancing, and partially necrotic mass in the left parietal area. The tumor invaded the posterior corpus callosum and was deemed unresectable. Biopsy led to the diagnosis of anaplastic astrocytoma, with molecular features of GBM: *MGMT*-unmethylated*, IDH1* WT, *EGFR* A298V/amp, *TERT* promoter mutation, *CDKN2A/B* deleted prompting an integrated diagnosis of GBM, WHO Grade 4. Conventional chemoradiotherapy (XRT 60 Gy with TMZ 75 mg/m^2^ daily) was administered. Thereafter, adjuvant chemotherapy (TMZ 150 mg/m^2^ × 5 days) was initiated. The patient required dexamethasone 2–4 mg daily for edema management.

During the first adjuvant TMZ cycle, worsening right hemiparesis occurred. He fell and sustained a fracture of the femoral neck. Bipolar hemiarthroplasy was performed. In the postoperative period, he developed deep venous thrombosis. Subsequently, in a rehabilitation facility, he contracted coronavirus disease 2019 (COVID-19) and was quarantined. These events led to a 6-week delay in his return to the clinic beyond the date of the second cycle of adjuvant therapy. By this time, he had become wheelchair bound and completely dependent due to dense right hemiplegia. Additionally, he developed inability to speak due to severe expressive aphasia. A repeat MRI showed the tumor increased to 6.6 cm with new areas of enhancement and edema. The interpretation of the study favored disease progression over pseudo-progression. Next-generation DNA sequencing (NGS) was obtained showing homozygous *CDKN2A* and *MTAP* loss, *TERT* c.-146 C > T, and *EGFR* p.A298V and amplification.

Because of the poor performance status and absence of meaningful second-line therapy, hospice was discussed with the patient but declined. Based on the results of NGS, the patient consented to and received the following treatment based on the rationale shown in Table 1: pemetrexed: 500 mg/m^2^ (q3w); bevacizumab: 7.5 mg/kg (q3w); candesartan 8 mg po bid.

**Table 1.** Therapeutic vulnerabilities of pemetrexed, bevacizumab, and candesartan (PBC) created by 9p21 loss of CDKN2A and MTAP.

Therapy	Genomic aberration	Synthetic lethal partner	Pathway
Pemetrexed	*CDKN2A*	DHFR	CDKN2A → p14^ARF^ → MDM2↓ → DHFR↓ → THF → Methylene-THF_TYMS → dTMP
			CDKN2A → p14^ARF^ → MDM2↓ → DHFR↓ → THF → Formyl-THF_GARFT → IMP → AMP
		GARFT	GARFT → IMP → AMP
		AICART	AICART → ZMP↓ → AMPK → MTOR↓→ HIF1A → VEGFA → KDR → PIK3CA → AKT → Survival
	*MTAP*	GARFT	GARFT → IMP → AMP
Bevacizumab	*CDKN2A*	VEGFA	CDKN2A → p14^ARF^ → MDM2↓ → p53↓ → VEGFA↓ → KDR → PIK3CA → AKT→ Survival
Candesartan	*MTAP*	PRMT5	MTA → PRMT5↓ → SAM → Symmetric_Dimethylation_Arginine

**Figure 2.**
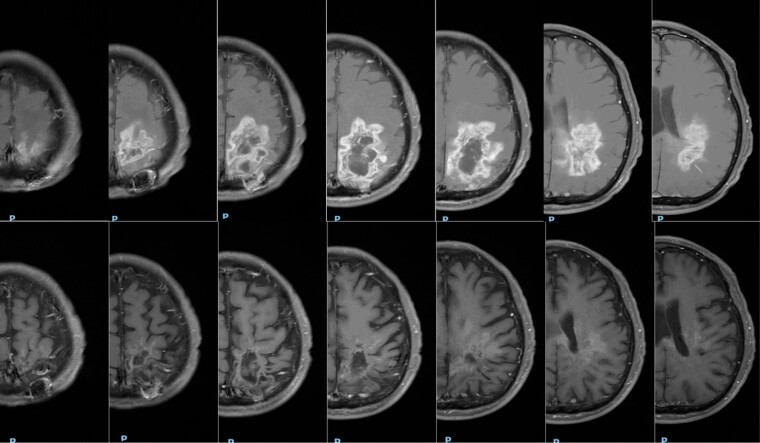
MRI axial images T1 + gadolinium contrast. Top: Baseline: imaging; Bottom: after 6 cycles pemetrexed-bevacizumab-candesartan: 5 months later. The follow-up study shows marked decrease in contrast enhancement and regression of disease.

The patient achieved an immediate and clinically significant response. After the first cycle, he recovered the ability to speak and ambulate with a walker, and dexamethasone was tapered. After 3 cycles, an MRI showed tumor regression with a greater than 50% decrease of all measurable enhancing lesions and a decrease in peritumoral T2/FLAIR signal. After an additional 3 cycles, the MRI shows continuing tumor regression. (Figure 2; additional coronal and sagittal comparisons are included in Supplementary File 1) No significant toxicity was encountered during treatment. Following 6 cycles of treatment, the MRI showed evidence of new disease representing disease progression.

## Discussion

SL targeting represents a novel method of killing cancer cells.^16^ The paradigm of synthetic lethality for cancers with homologous recombination repair deficiency has led to regulatory approval of poly-ADP ribose phosphorylase (PARP) inhibitors in 4 solid tumor malignancies.^17^ Concurrently, systematic SL interrogation is being conducted in a number of initiatives.^18^ The discovery of variable chemotherapy responsiveness arising from SL vulnerability derives from understanding signaling pathway consequences of specific genomic abnormalities and how they intersect with classical drug targets. As such, SL provides a basis for understanding variable antitumor efficacy and identifying which patients are most likely to benefit from a given cytotoxic approach. In some cases, SL provides a basis for understanding the “exceptional responder” phenomenon.^19^

Many cancers offer multiple SL targets thereby illuminating a strategy for combination therapy design. By exploiting as many SL opportunities as possible, the complex proteogenomic network underlying cancer can be targeted at its most vulnerable nodes leading to disease collapse. *CDKN2A* loss is unique in providing several synthetic lethal targets that can be targeted by a single drug. Additionally, SL vulnerability of *CDKN2A* and *MTAP* both converge on GARFT making it possible to target two gene abnormalities with one drug, that is, hitting 2 birds with 1 stone. Moreover, both tumor suppressor genes have other SL partners, including VEGFA and PRMT5, defining a dysfunctional network with multiple vulnerabilities that confer treatment opportunities for network takedown.

Traditionally, oncologic thinking invokes the necessity of causing normal tissue toxicity based on the similarity of normal and malignant tissues. However, normal tissues lack somatic cancer-related abnormalities and do not have SL vulnerabilities. Therefore, SL targeting defines a strategy with lethal antitumor effects that can be executed with little or no consequence for normal tissues providing an exceptionally large therapeutic index. An exception would be for patients who carry a germline *CDKN2A* mutations, effectively conferring enhanced normal tissue susceptibility to pemetrexed. Or the absence of SL vulnerability in cancer with *CDKN2A* wild type, *9p21* intact presents a situation of limited cytotoxicity from pemetrexed for malignant tissue, thus making the therapeutic index close to zero. By contrast, network targeting that exploits *somatic* SL vulnerabilities offers a theoretical framework for capitalizing on a particular cancer’s unique vulnerabilities *without* normal tissue toxicity. Remarkably, the loss of specific tumor suppressor genes in GBM identifies common subsets of patients (e.g. *CDKN2A, MTAP, PTEN, RB1, NF1, TSC1*) creating testable SL treatment hypotheses for a majority of patients with this malignancy.

We acknowledge that either bevacizumab or pemetrexed alone might have produced the observed response. However, it is likely that the synthetic lethal vulnerabilities in the tumor’s network conferred a response to *both* agents. Thus, the deployment of the combination capitalizes on a dual therapeutic opportunity and in this way could achieve synergy in producing the observed response. The alternative of treating with only single agents neglects network nodes capable of mediating resistance and disease progression. Mechanistically, the autocrine activation of KDR **→**Pi3K/AKT signaling, arising from VEGFA upregulation caused by p53 takedown, could activate survival, DNA repair, and epithelial–mesenchymal transition (EMT) pathways that oppose the efficacy of antimetabolite chemotherapy. However, VEGFA targeting with bevacizumab defeats, if only partially, these resistance mechanisms to enhance the cytotoxic effect of pemetrexed. In this way, network targeting combination therapy (NTCT) overcomes adaptive resistance mechanisms that exist in complex and heavily resourced proteogenomic networks. As such NTCT provides a theoretical basis for combinatorial treatment design that surpasses the limitations of single agents. Potentially, co-targeting EGFR and TERT mutations might have further improved the time to network adaptation and disease progression in this patient.

In conclusion, *9p21* deletion represents a chromosomal syndrome with a distinct aggressive biology and inferior survival, but one that appears uniquely sensitive to PBC, thus turning a negative prognostic biomarker into a positive predictive biomarker. NTCT of key nodes in *9p21*-deleted cancer effectively turns lethal disease drivers against themselves. Remarkably, SL relationships identify exceptional responsiveness to specific chemotherapy agents and offer a new understanding of why some patients achieve rapid and durable remissions. In some situations, cancer’s capacity to re-establish homeostasis in the face of treatment, that is, robust perfect adaptation, is counterbalanced by synthetic lethal or synthetic sick vulnerabilities which offer potential treatment options for patients. Not surprisingly, *CDKN2A* and *MTAP* loss is emerging as an important predictor of pemetrexed benefit in solid tumor oncology. This case report extends the promise of *9p21* deletion as a biomarker for therapy selection to patients with GBM. The utility of PBC deserves to be tested prospectively in GBM patients with this commonly encountered genomic aberration.

## Supplementary Material

vdad162_suppl_Supplementary_File
