# Supplementary figures and images for: Network targeting combination therapy of synthetic lethal vulnerabilities in 9p21-deficient glioblastoma: A case report
Source: Neurooncol Adv. 2023 Dec 10;6(1):vdad162. doi: 10.1093/noajnl/vdad162 (PMC10771271; doi:10.1093/noajnl/vdad162)

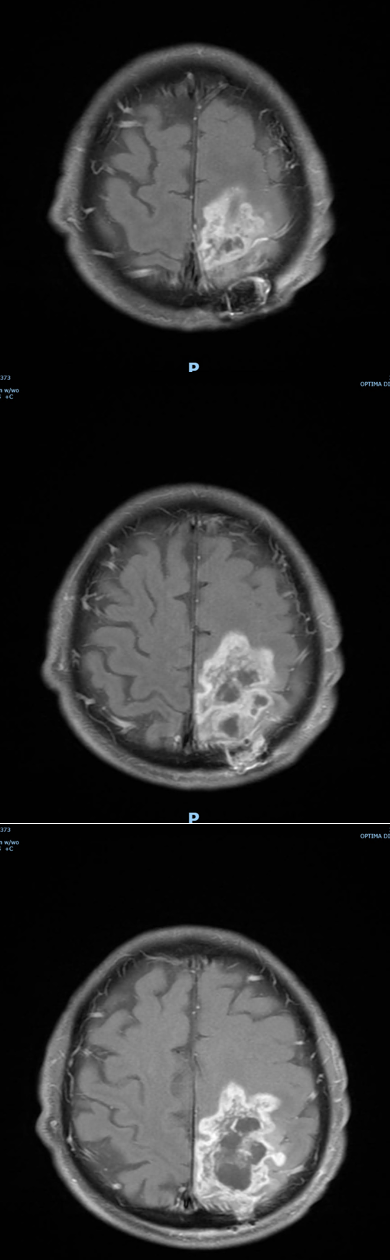

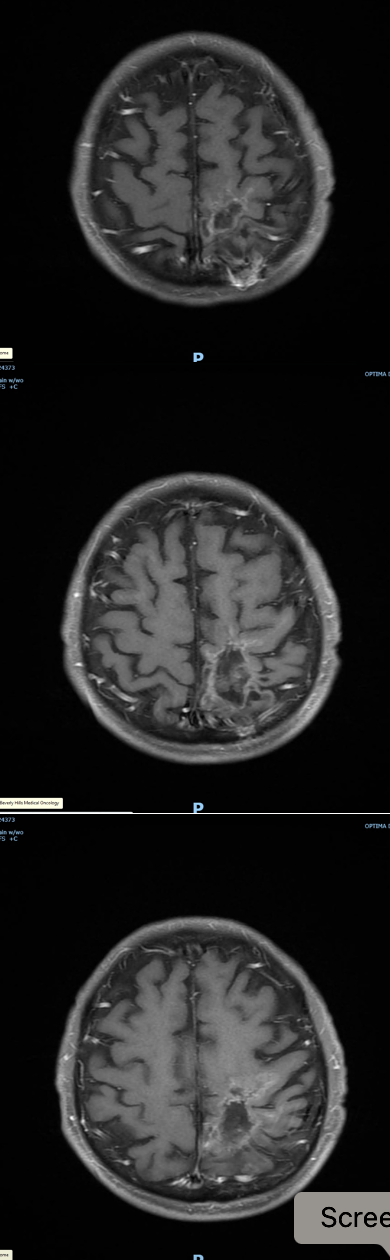


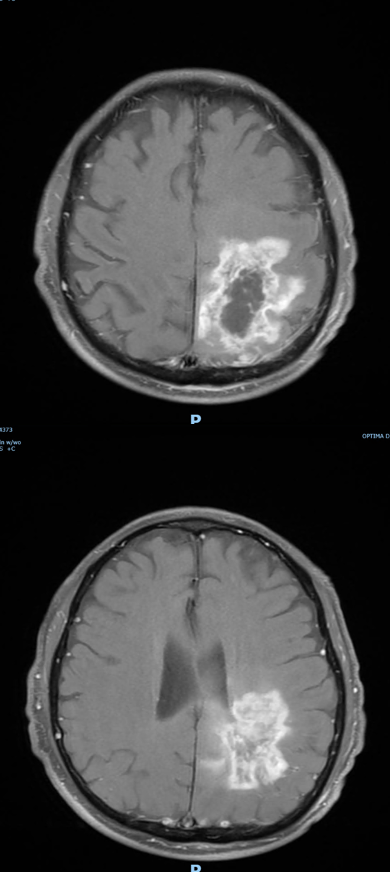

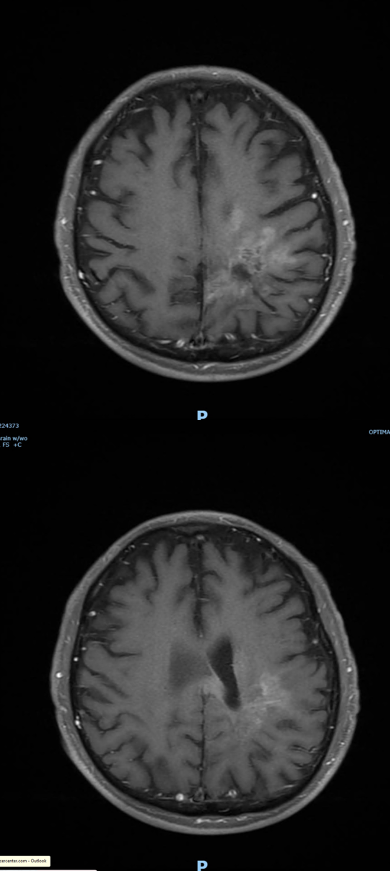


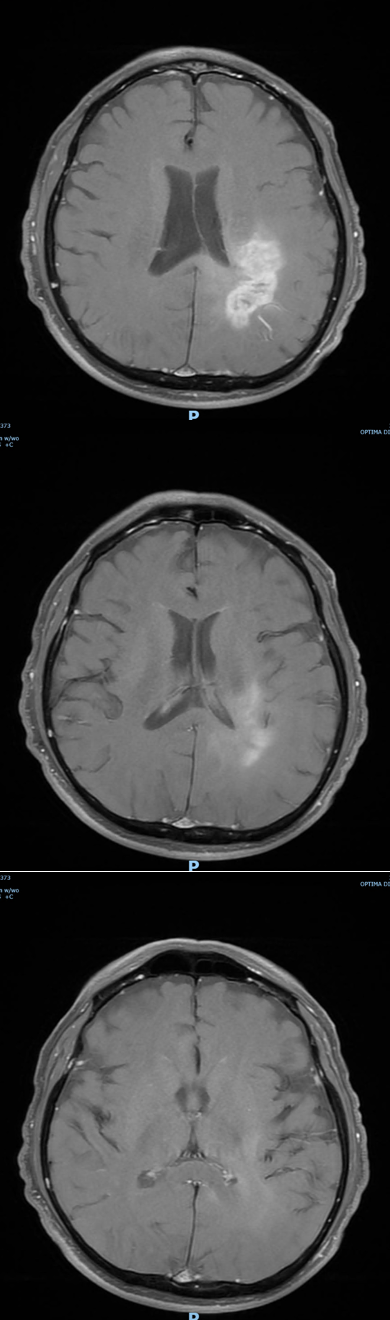

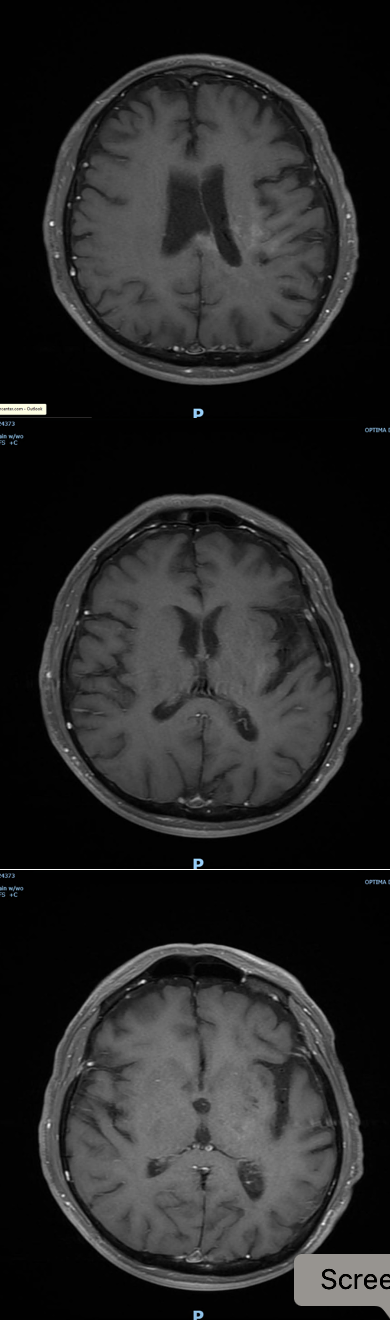


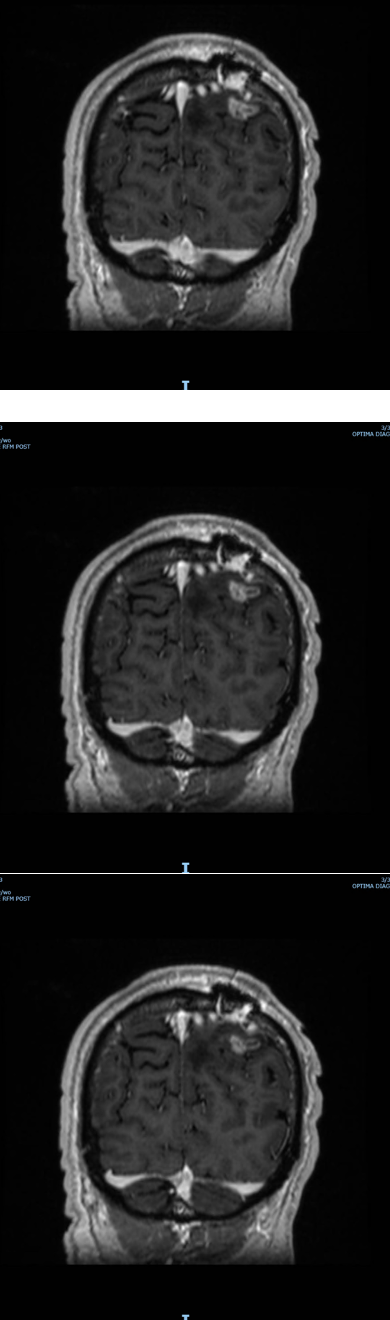

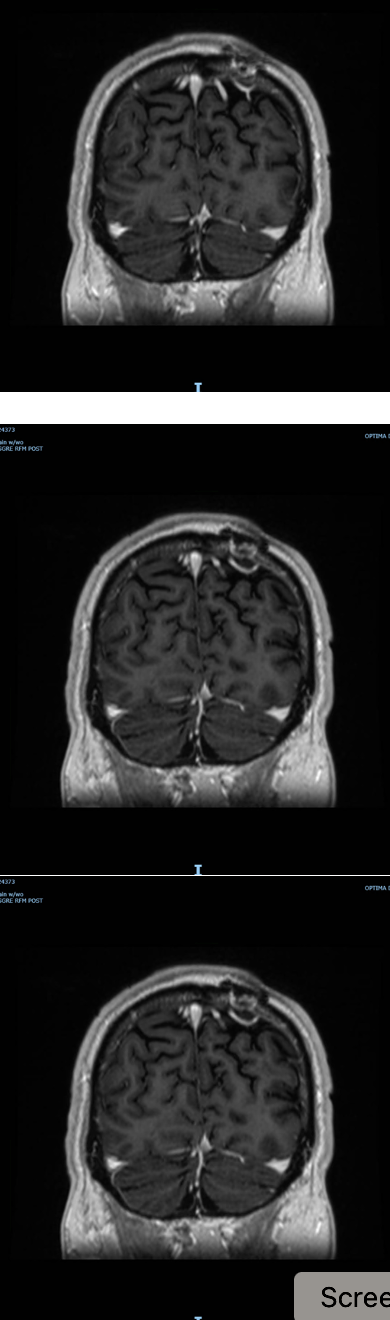


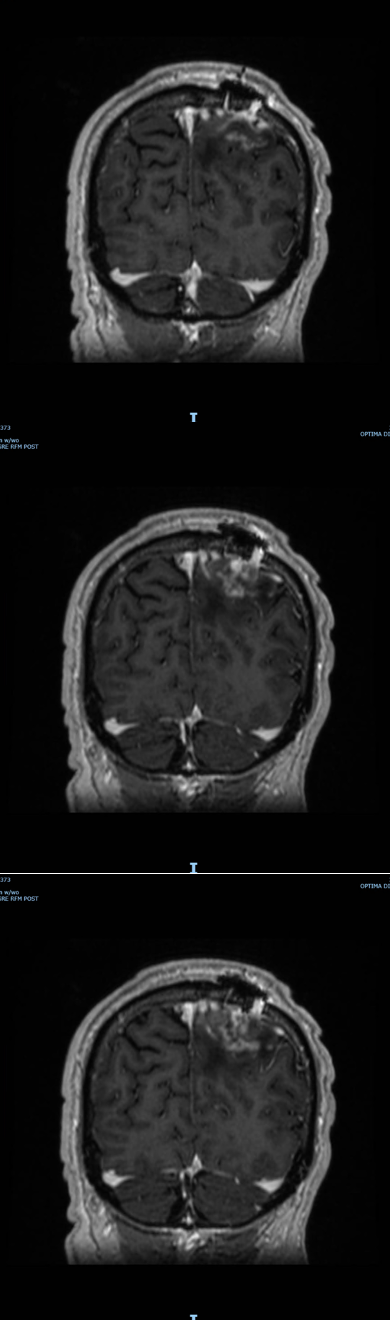

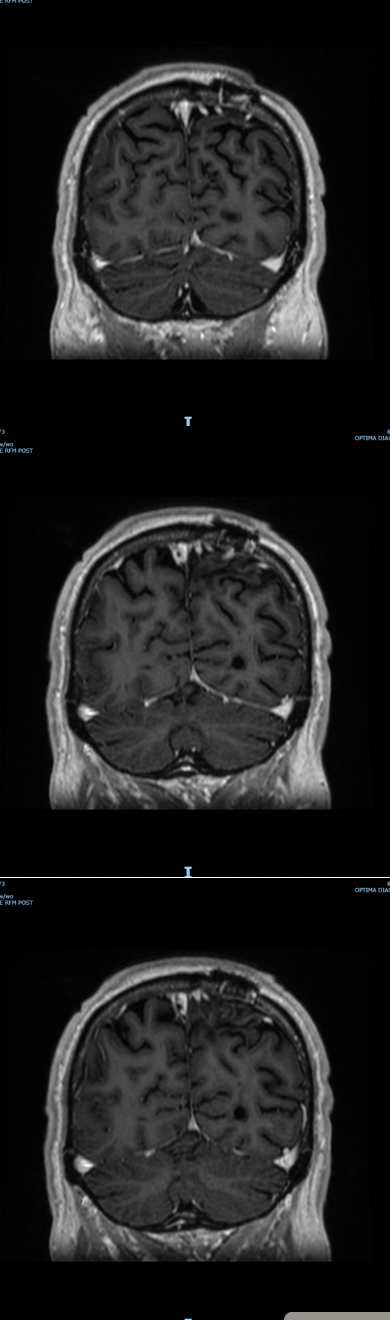


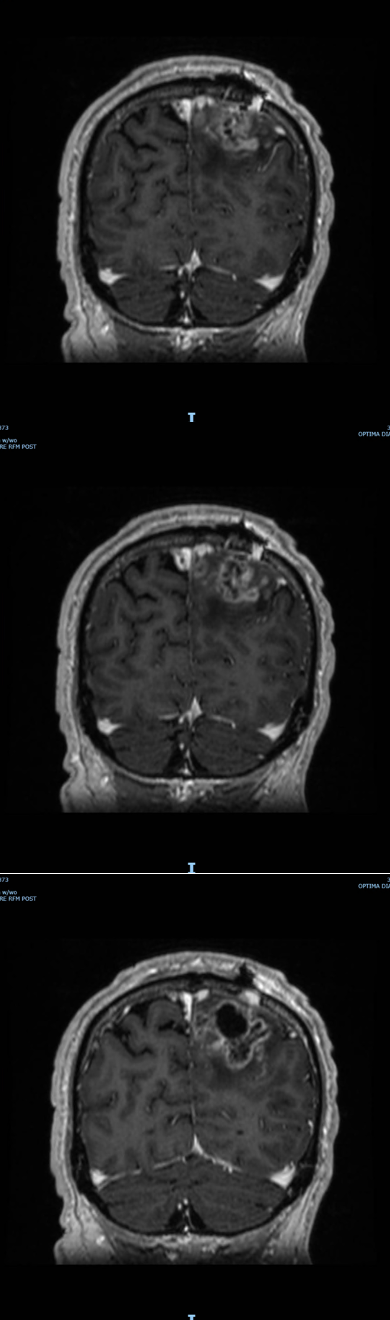

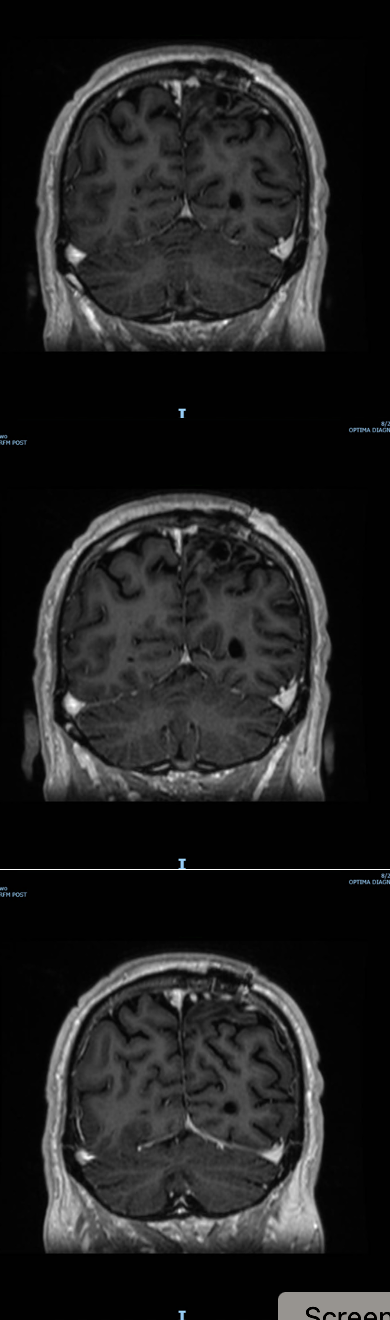


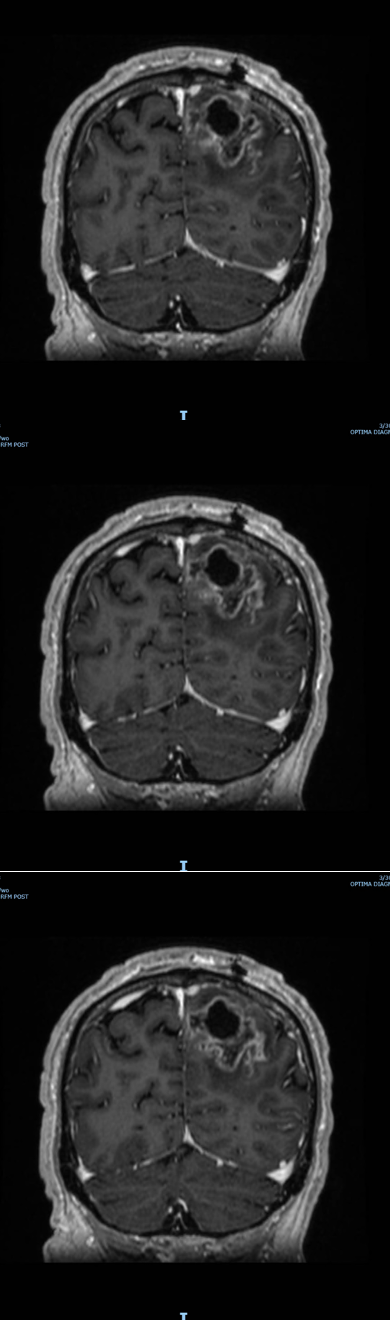

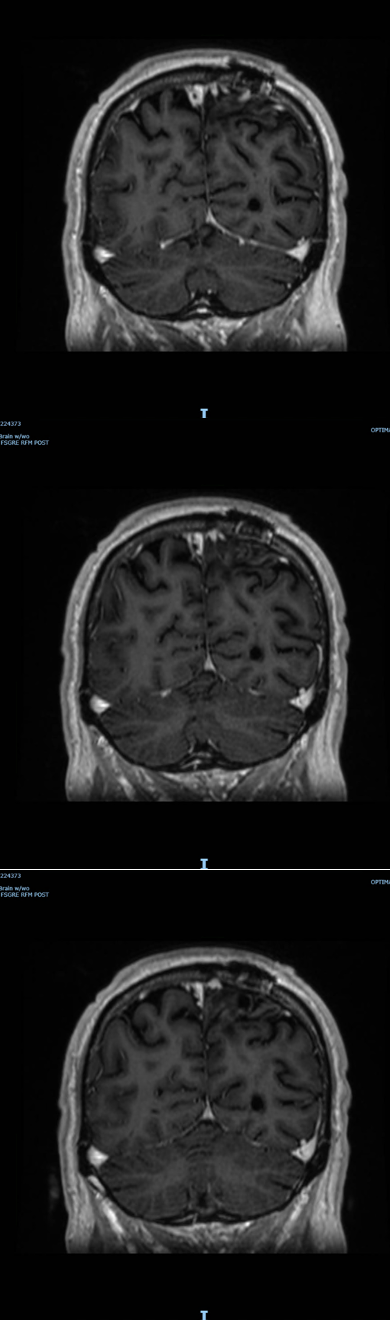


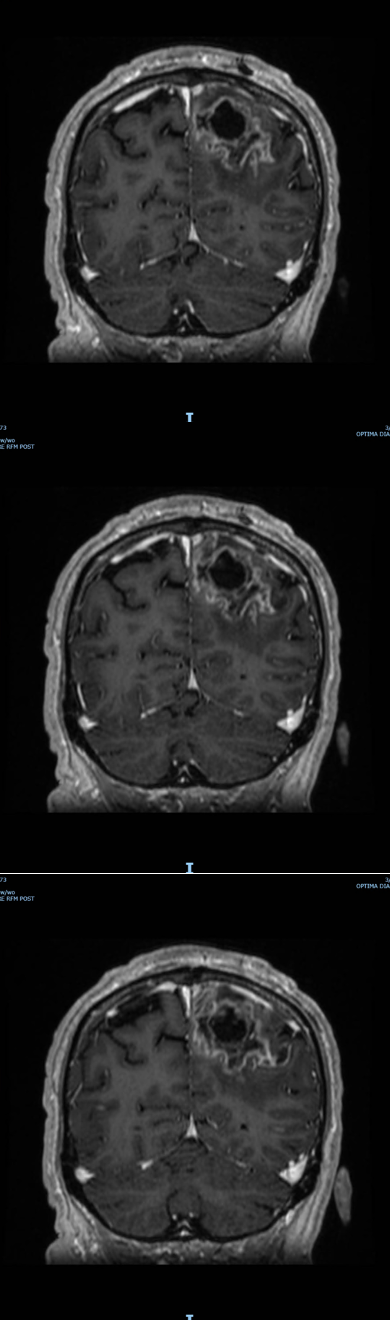

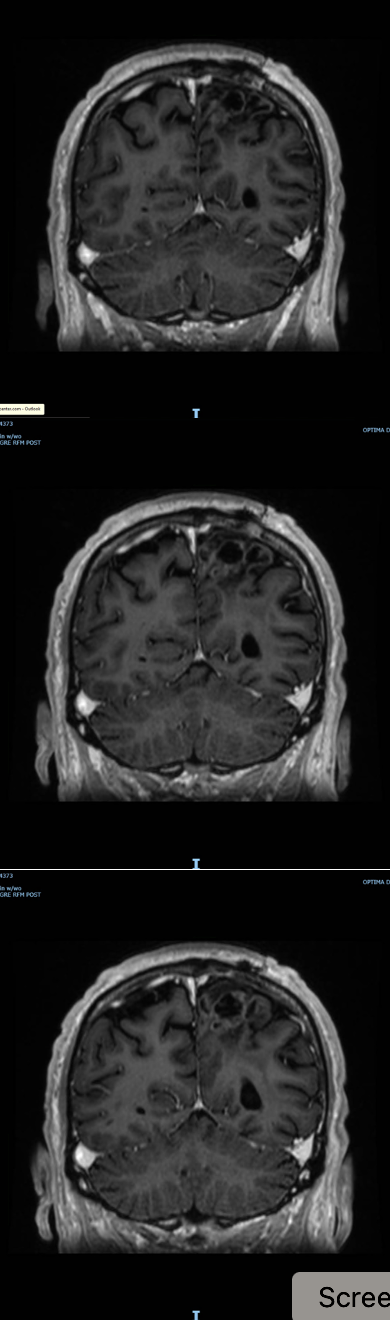


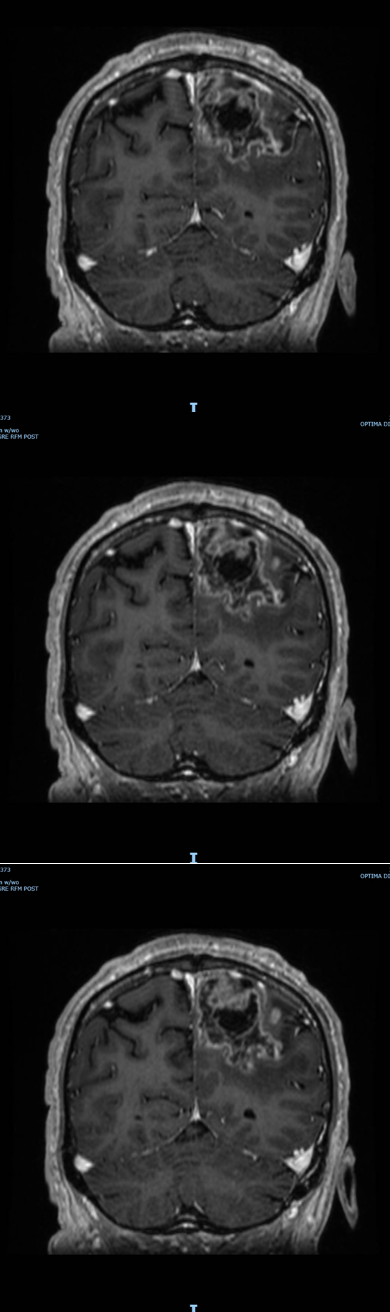

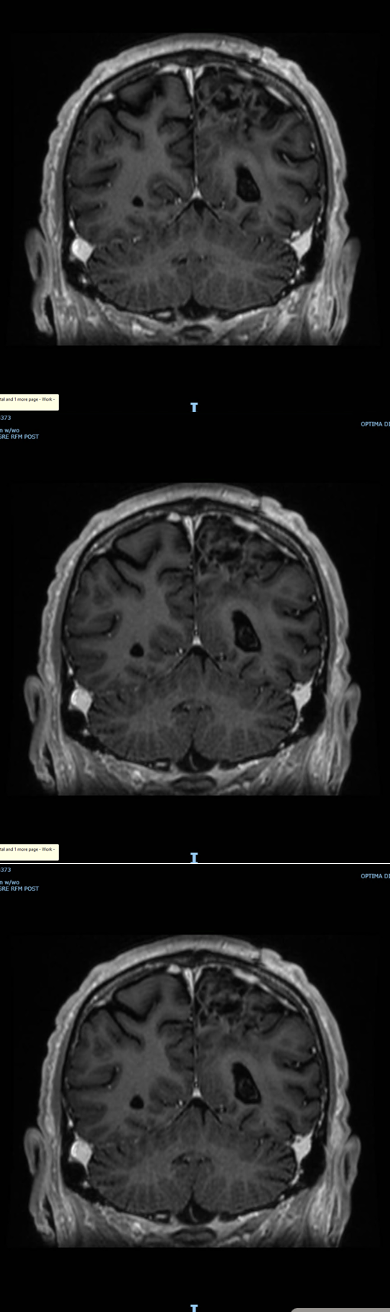


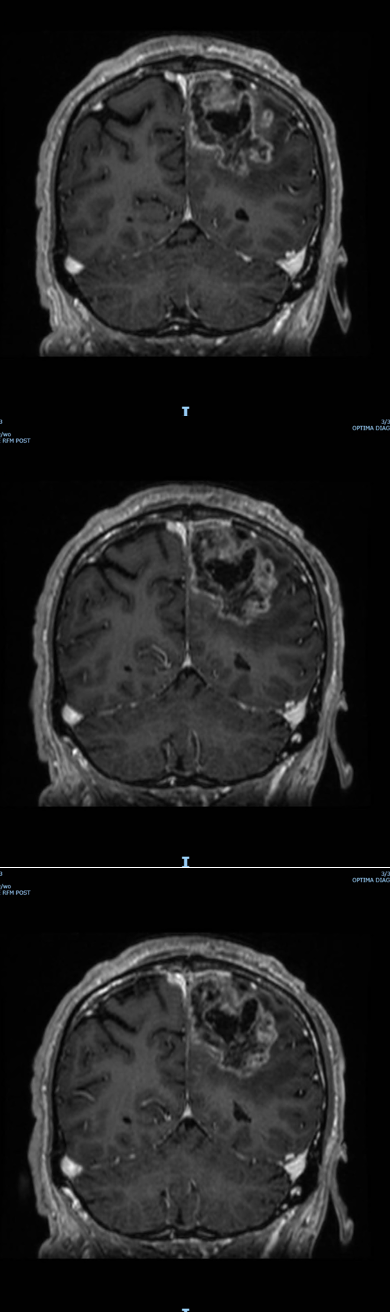

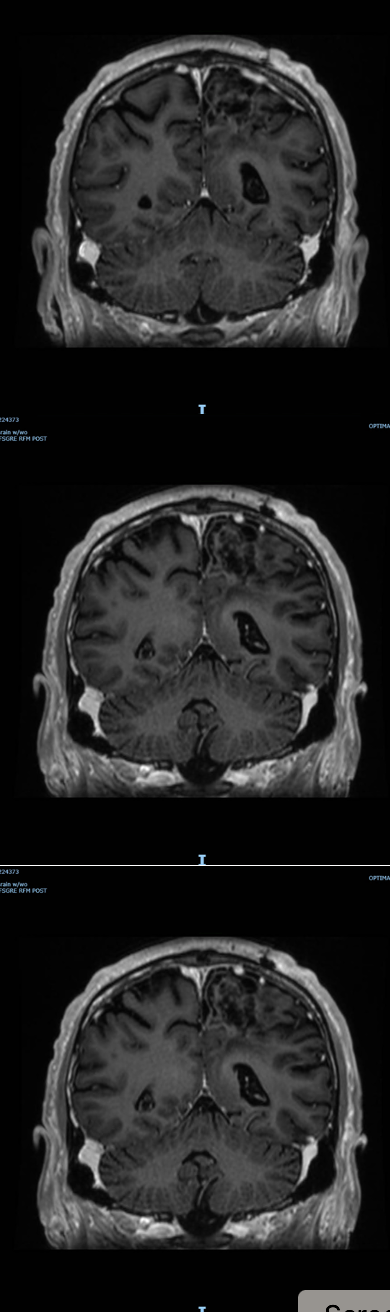


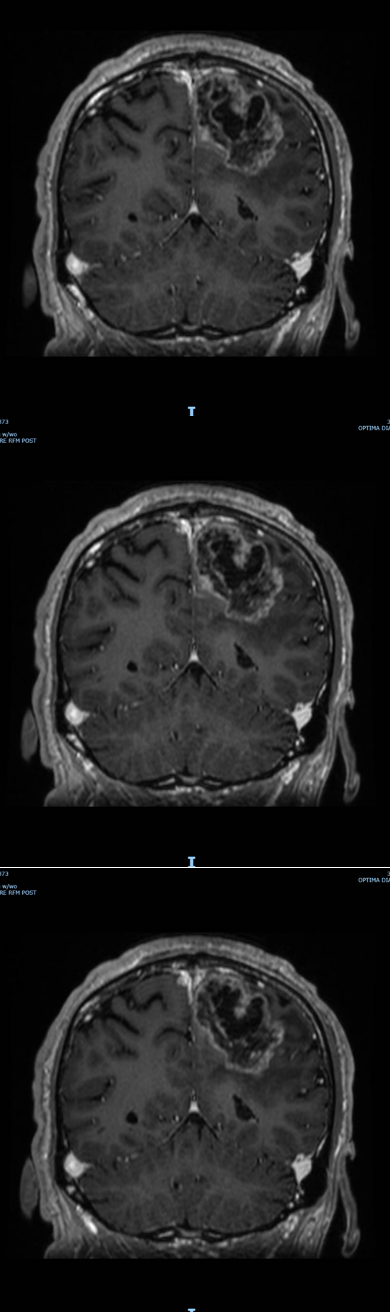

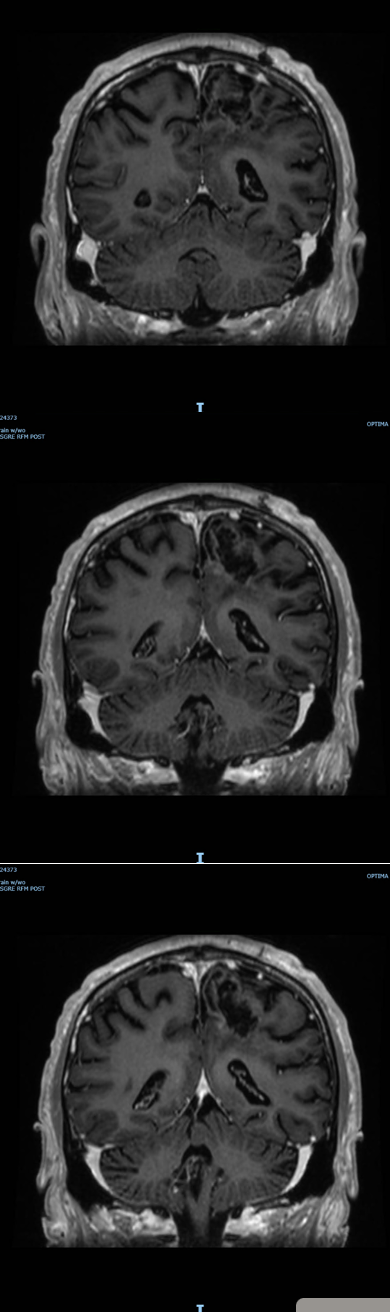


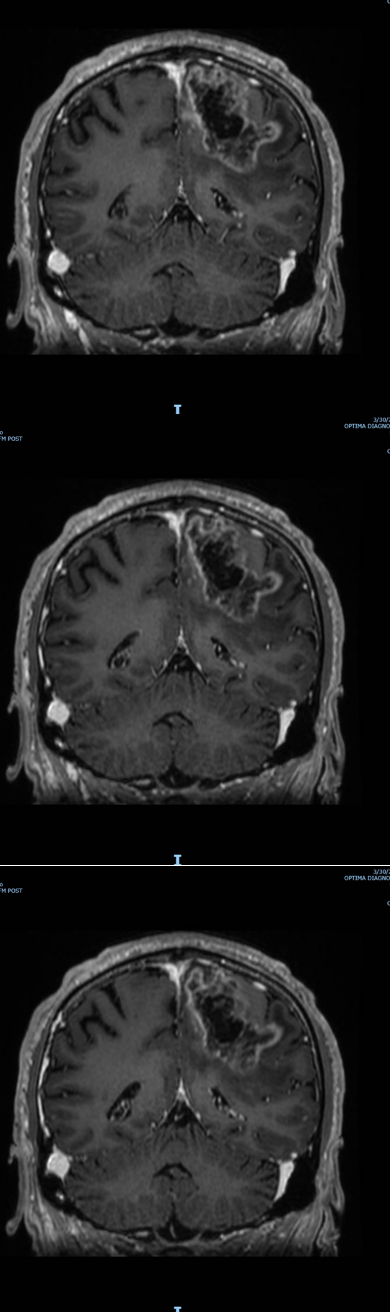

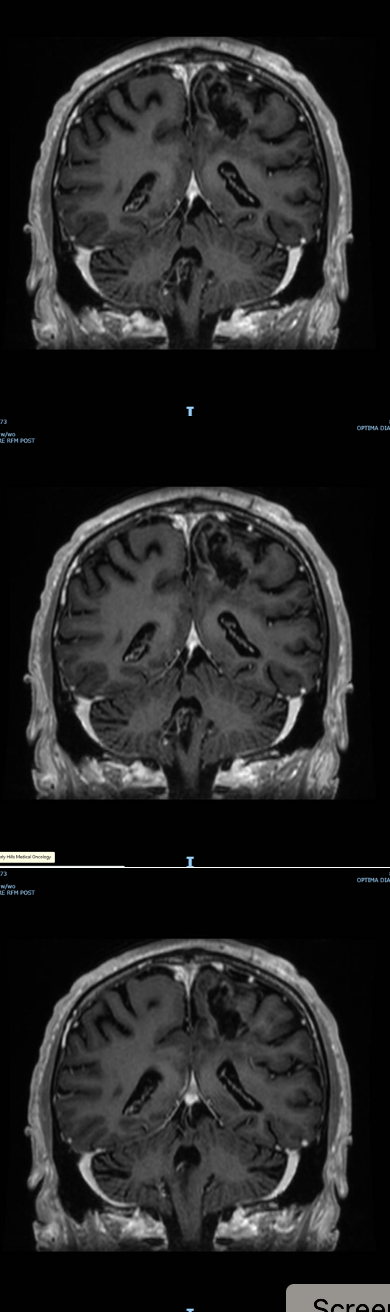


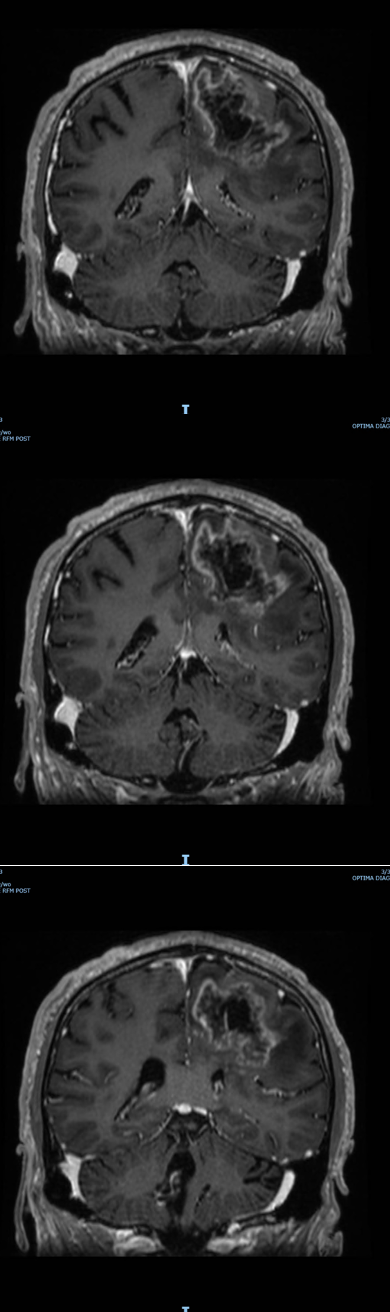

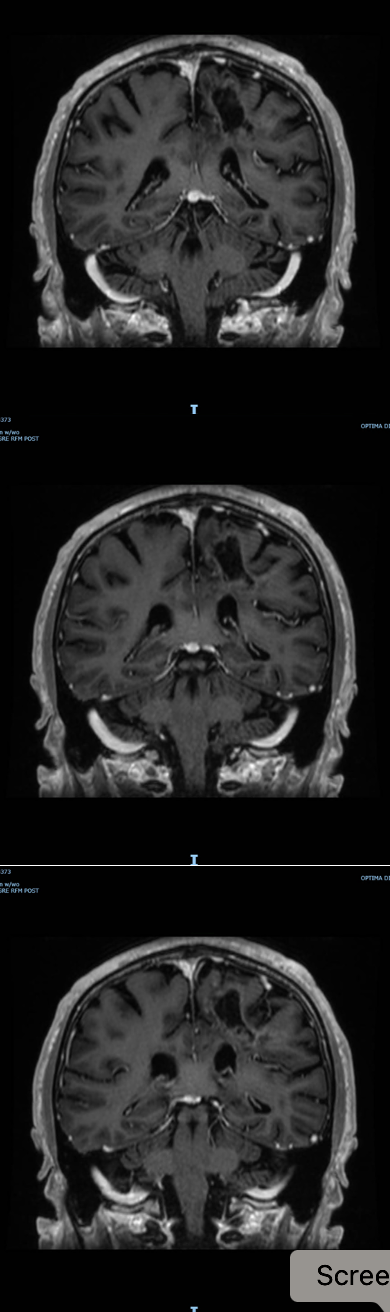


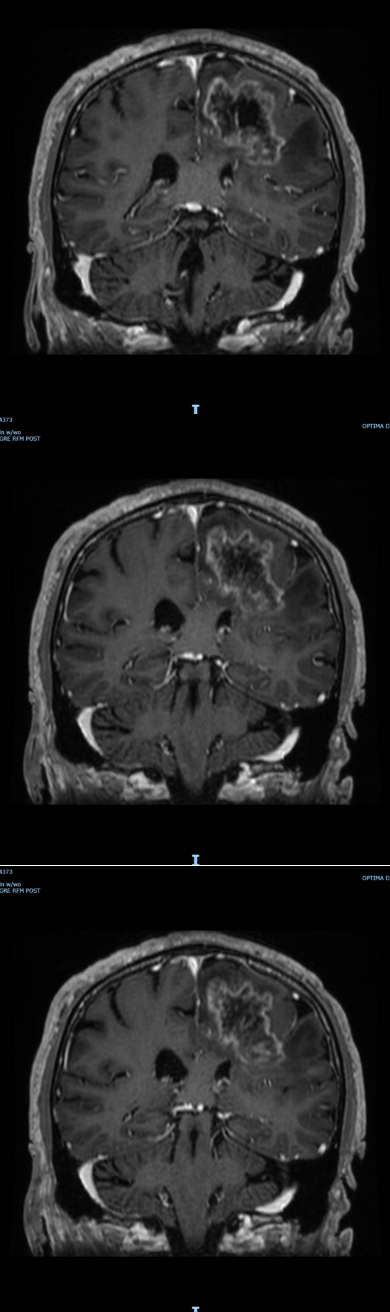

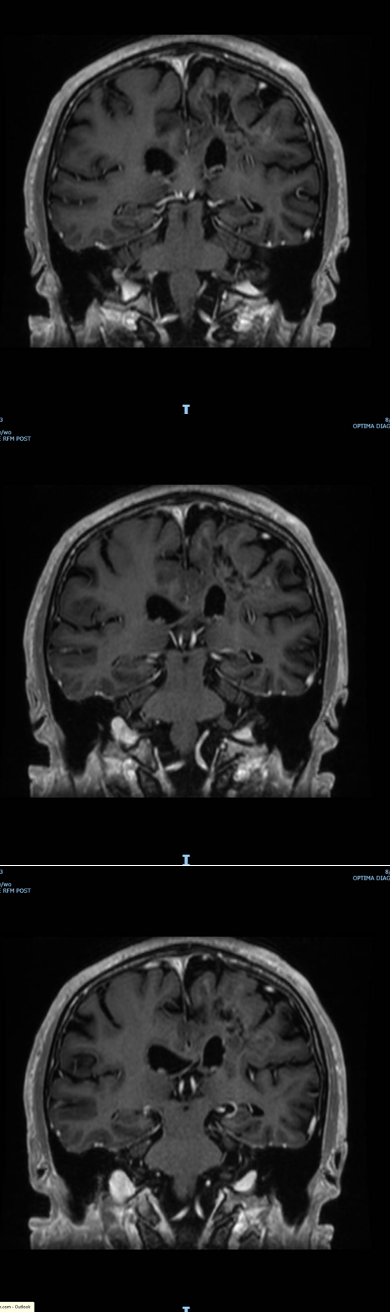


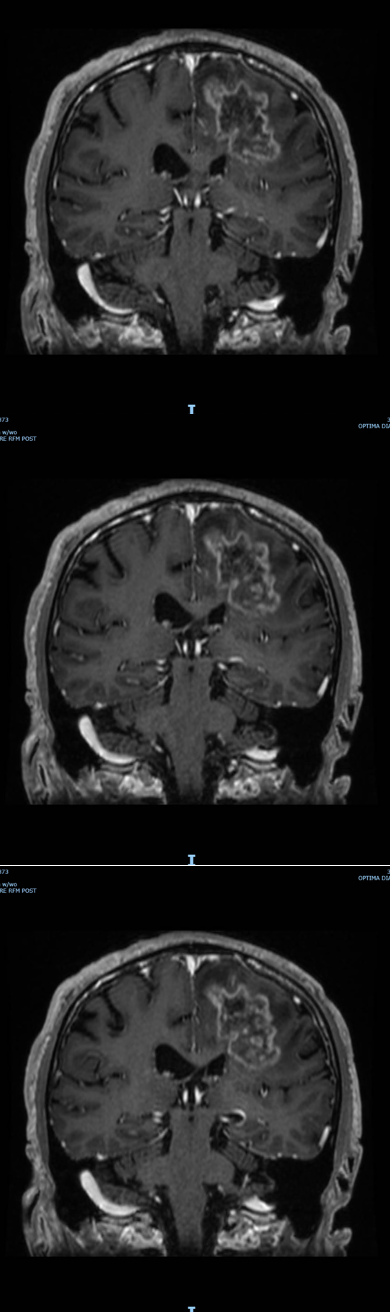

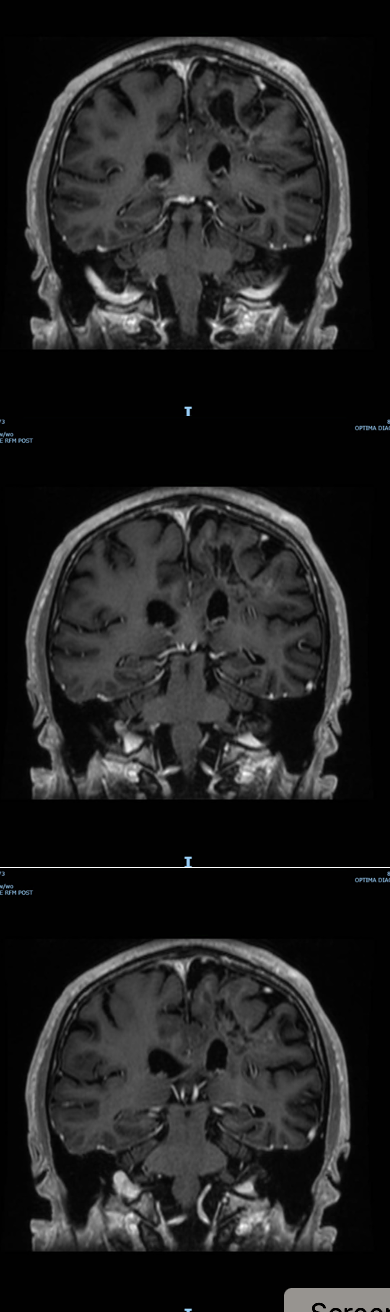


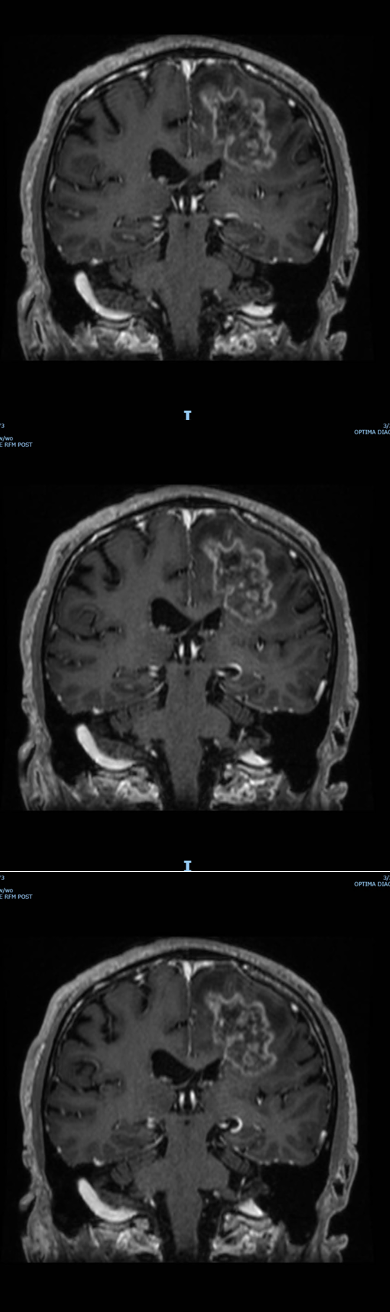

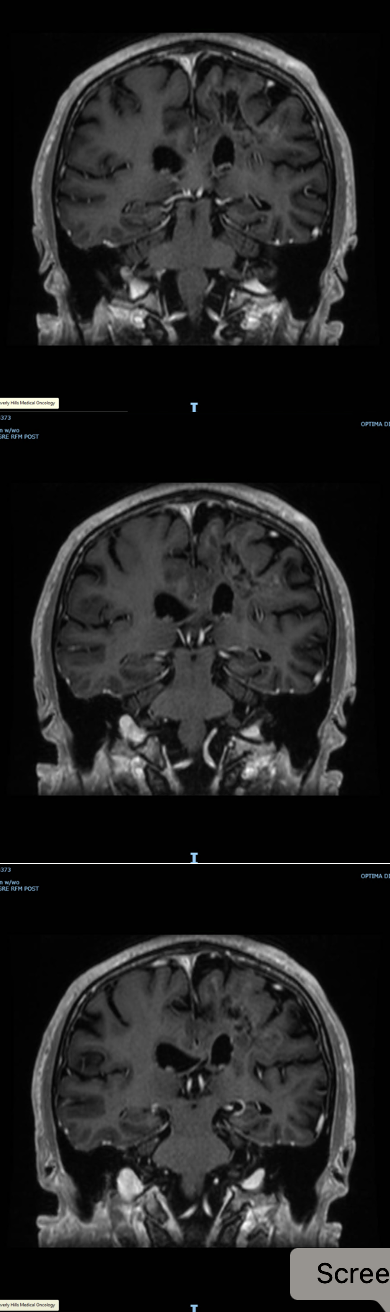

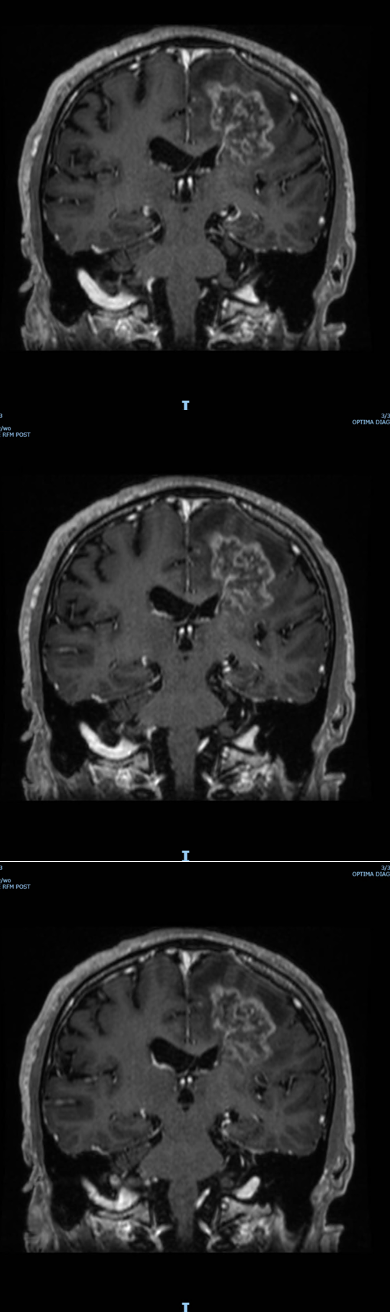

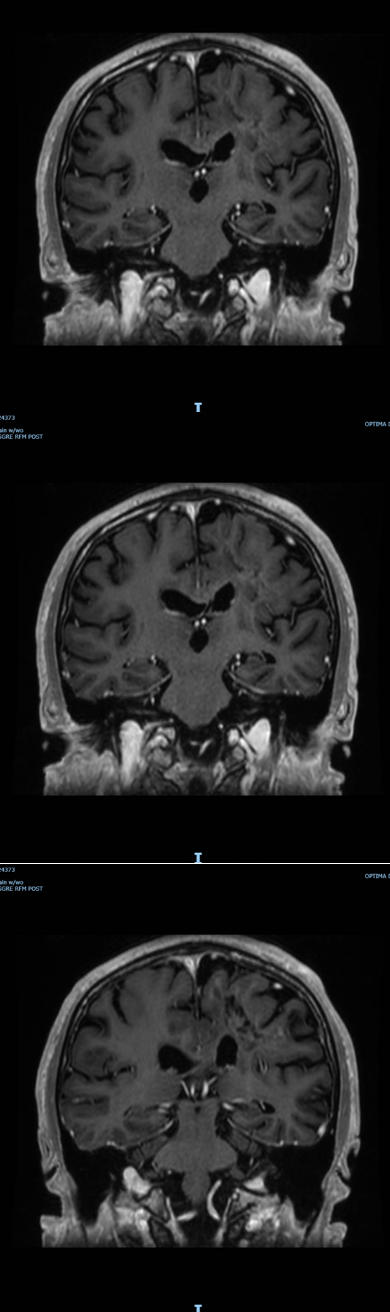

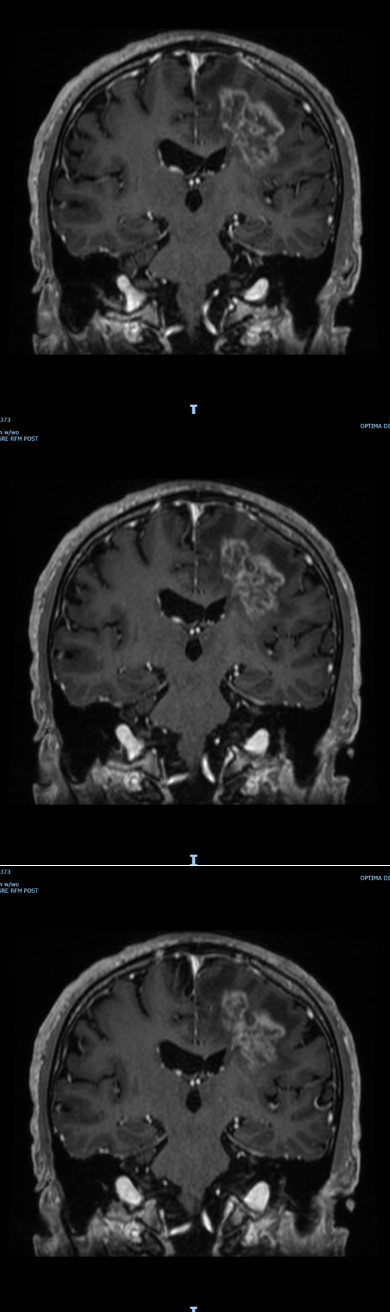

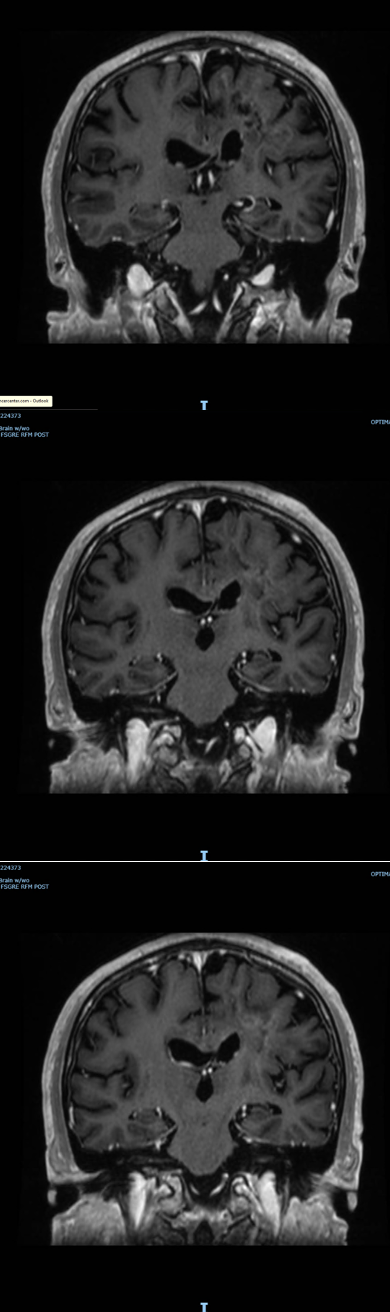

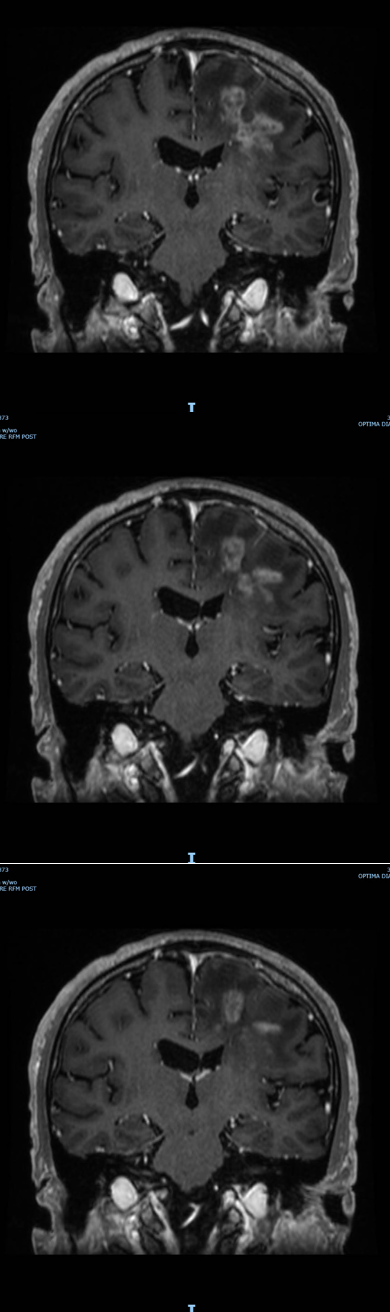

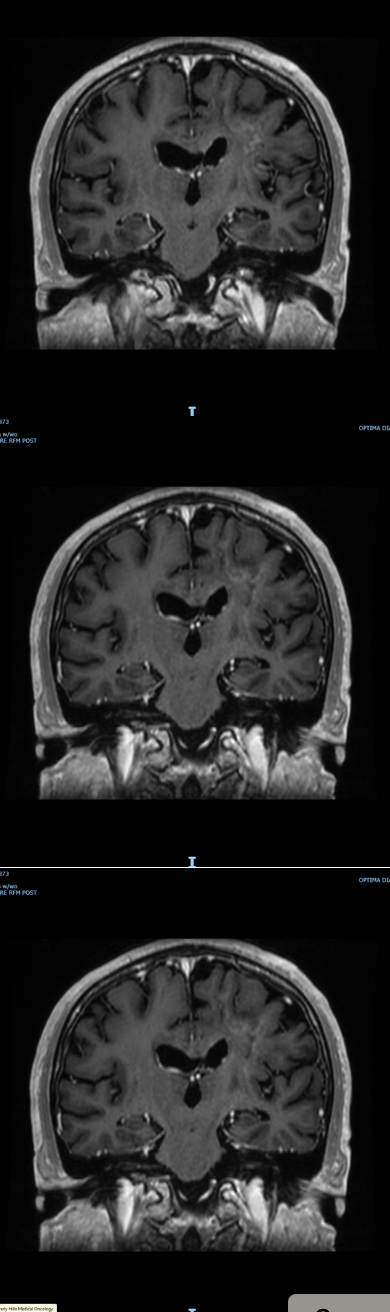

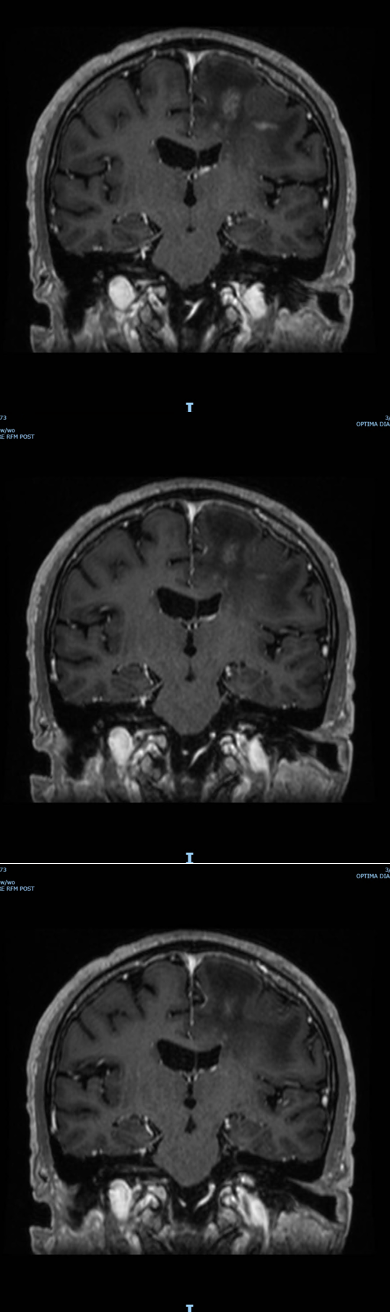

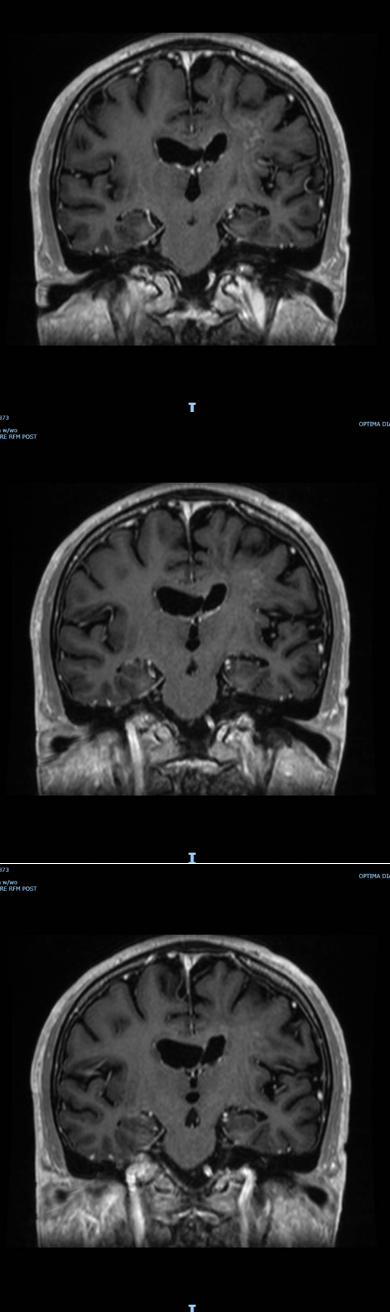

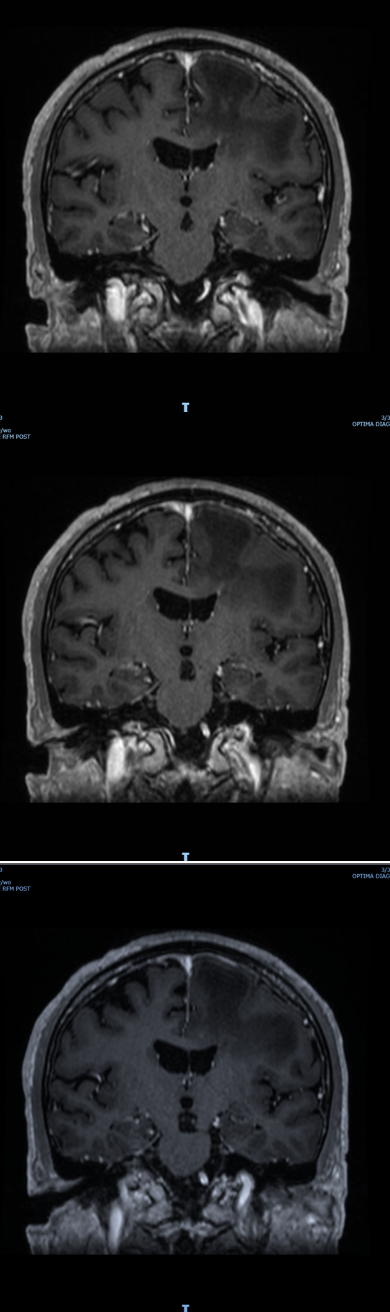

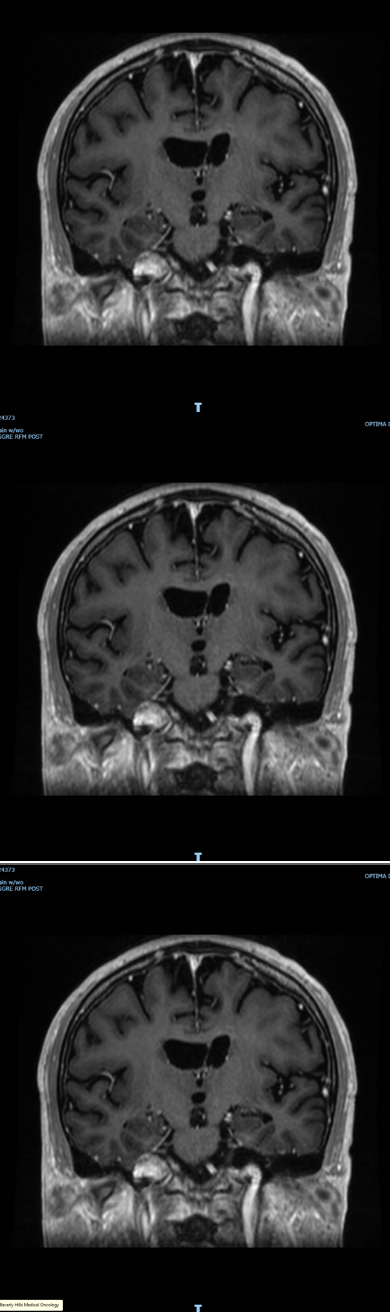

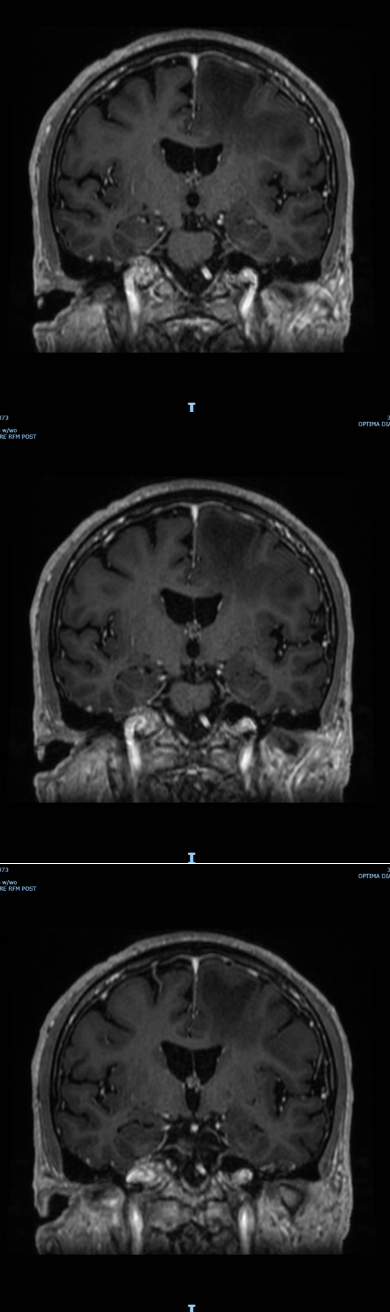

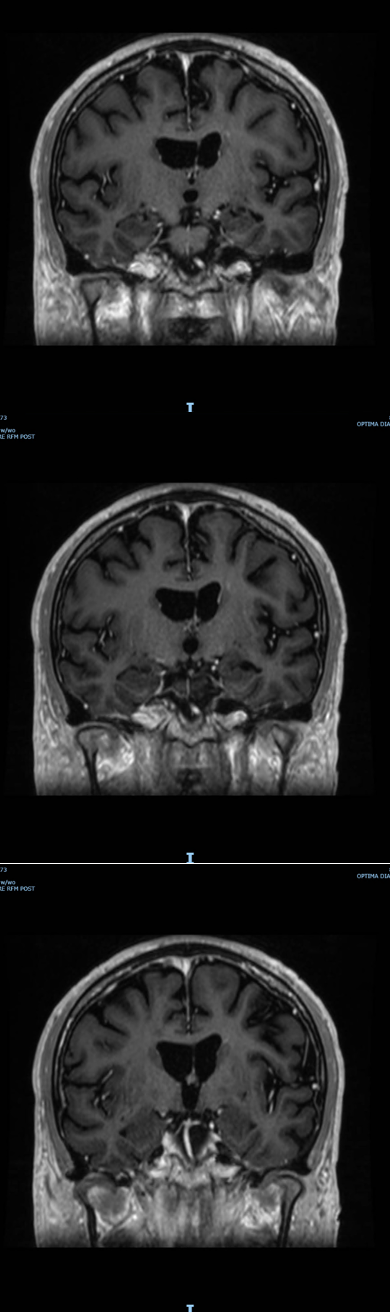

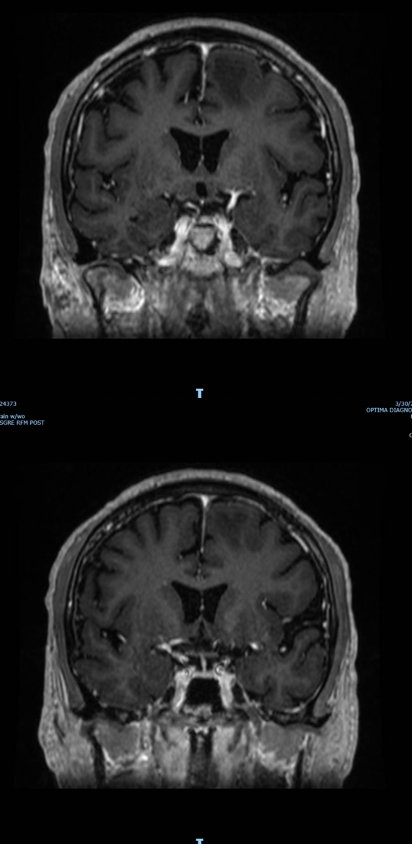

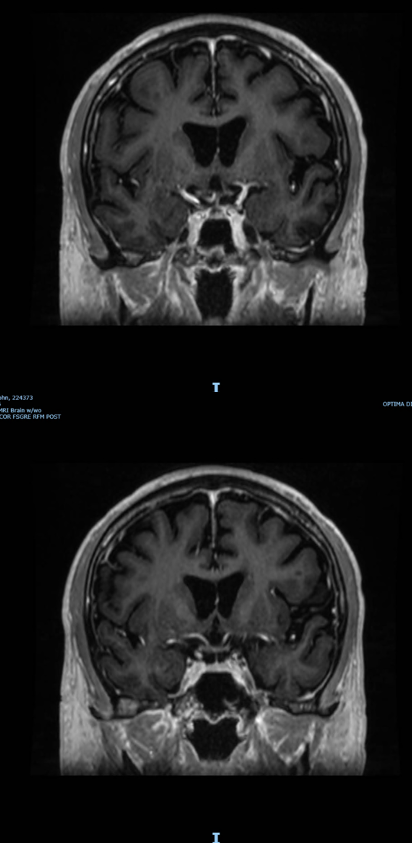


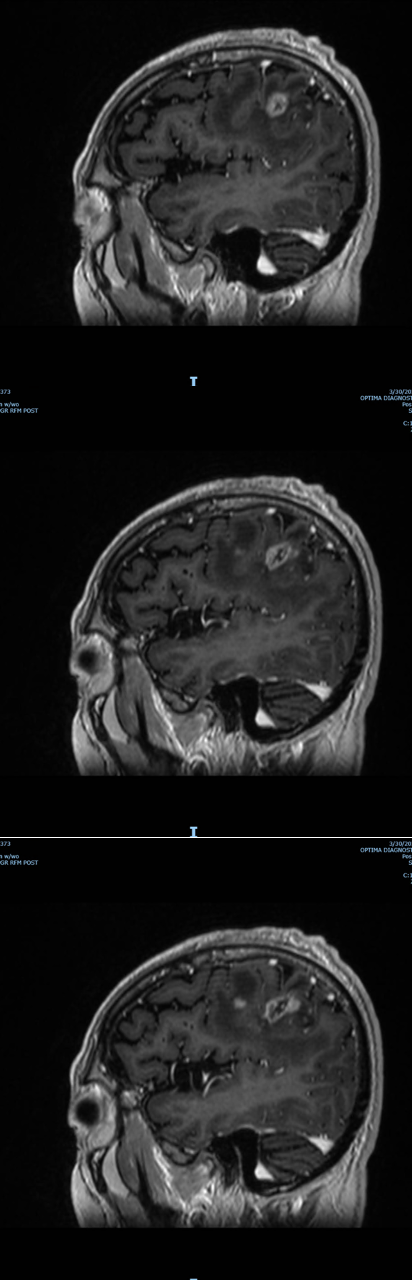

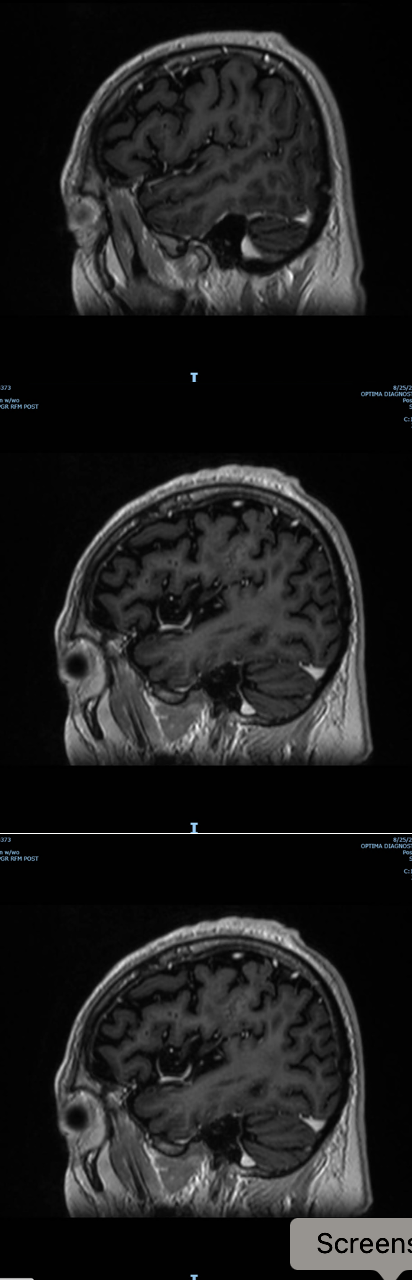

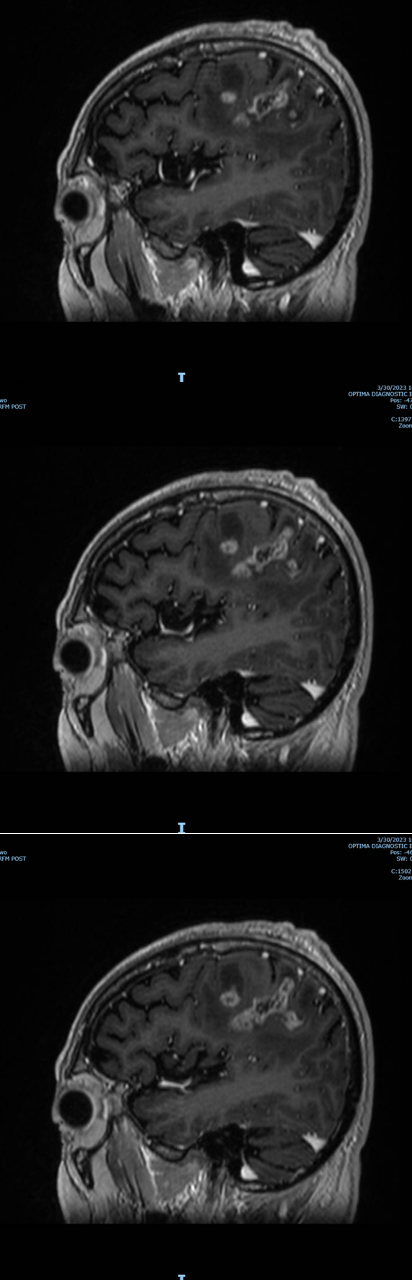

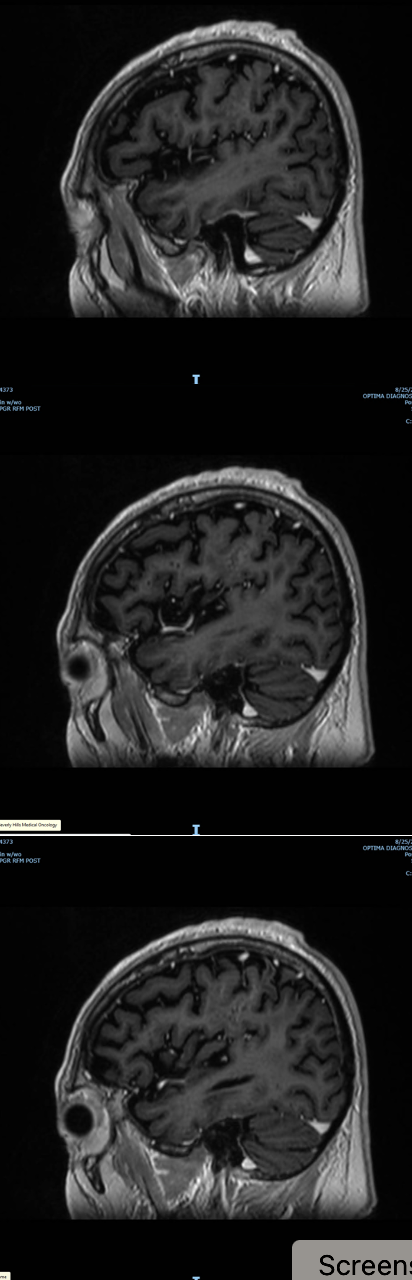

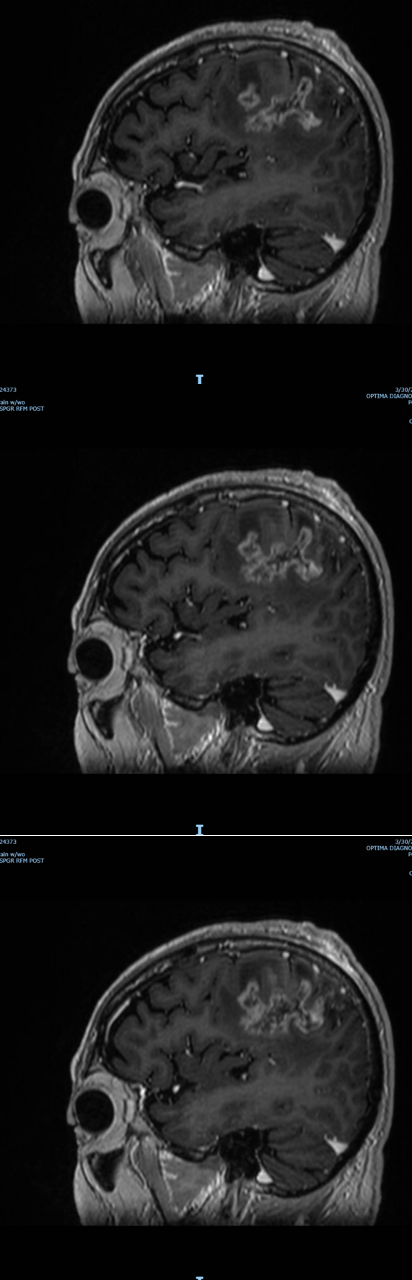

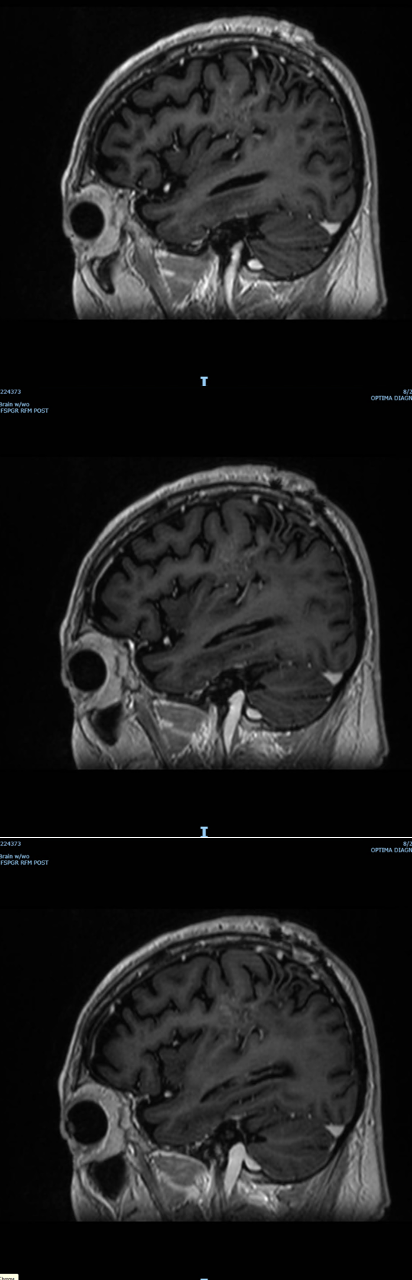

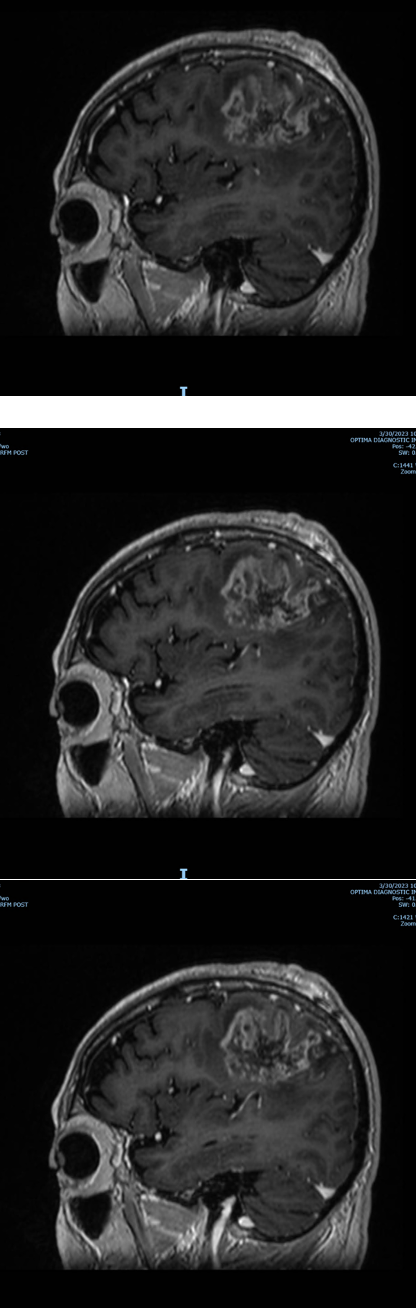

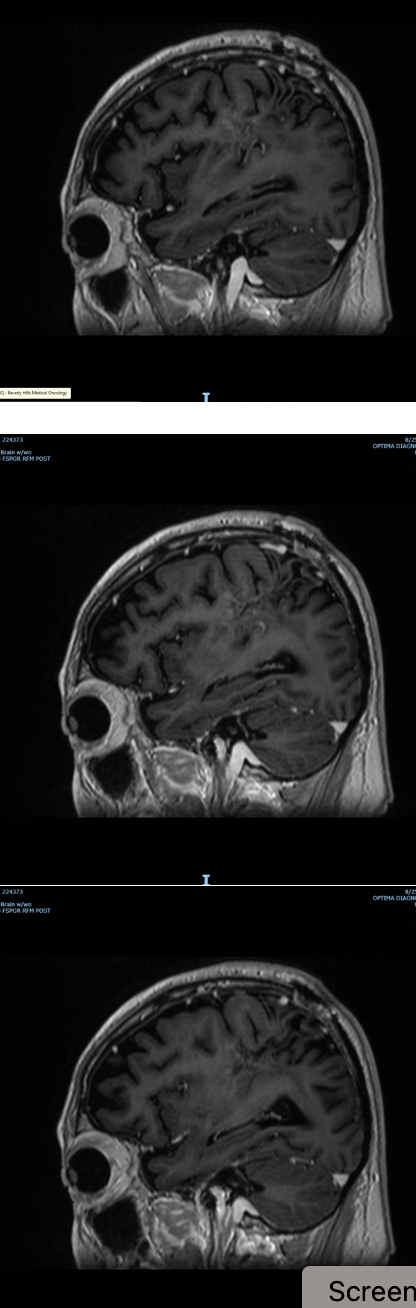

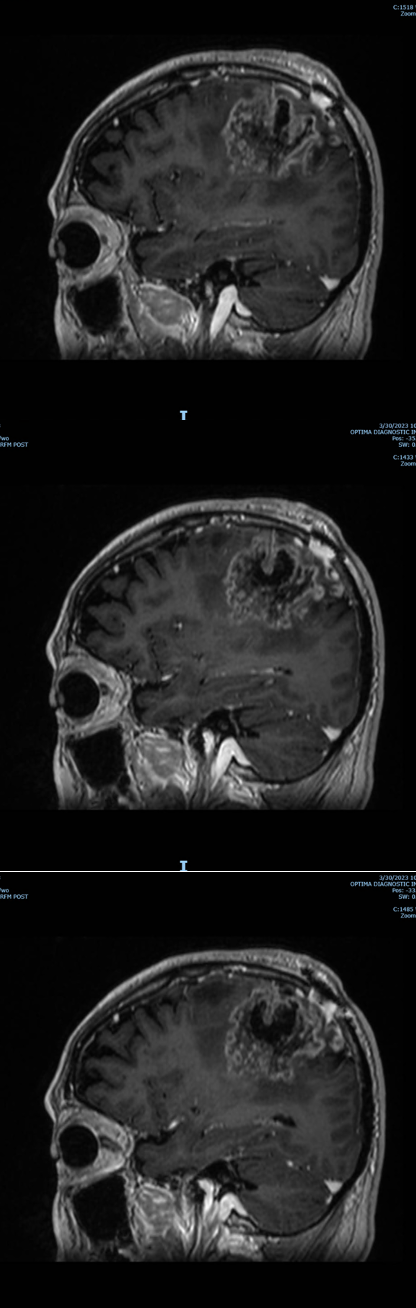

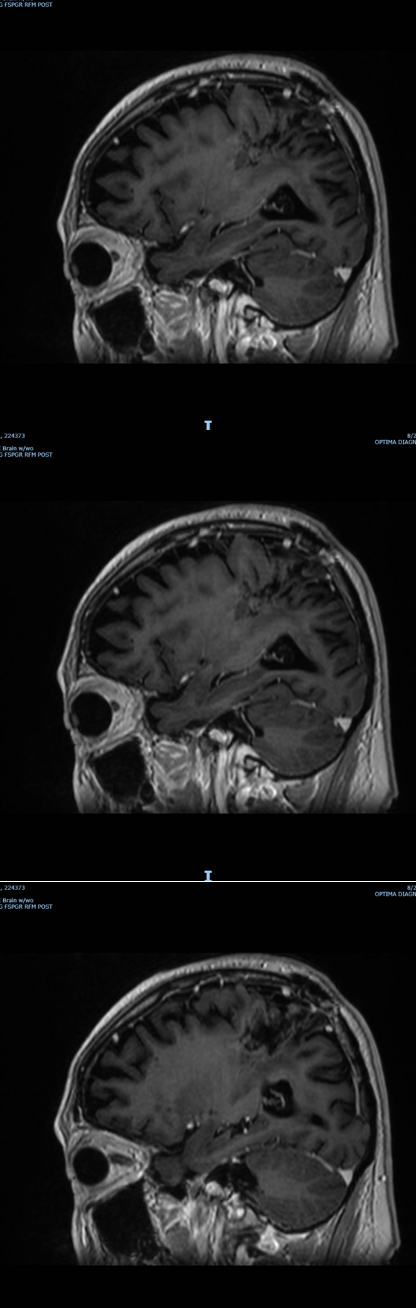

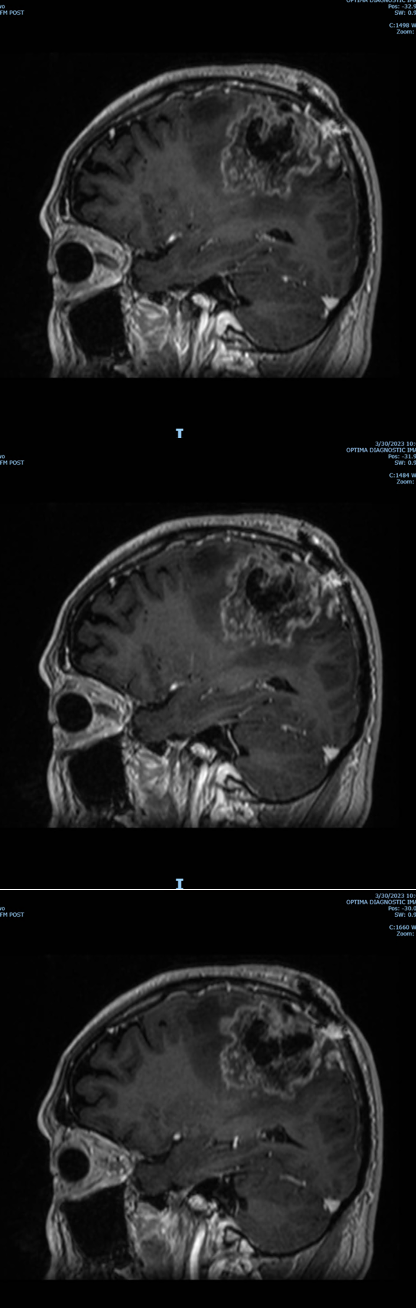

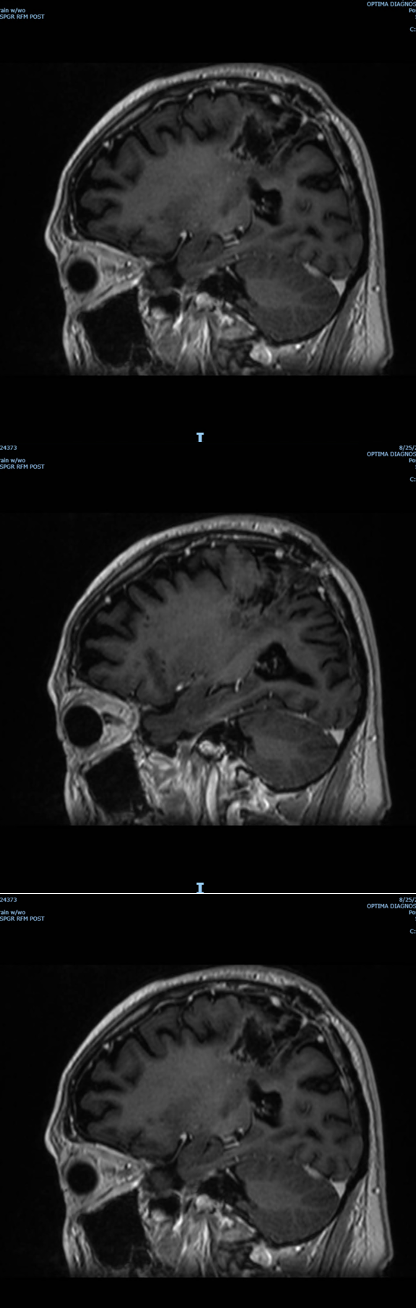

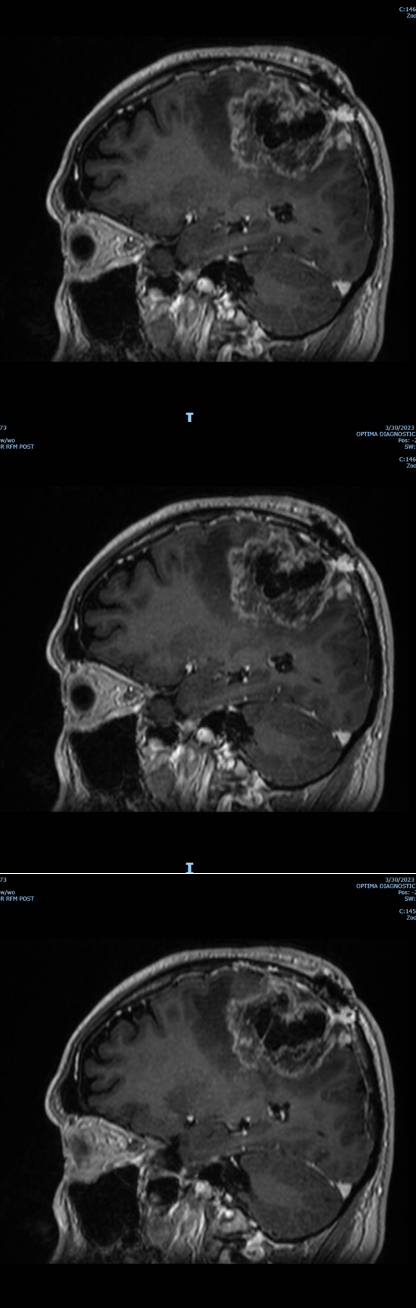

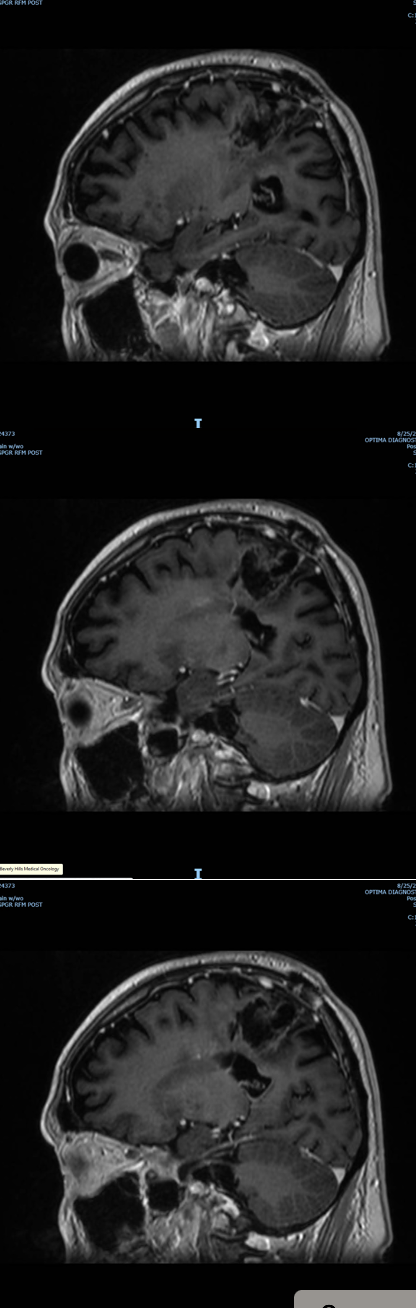

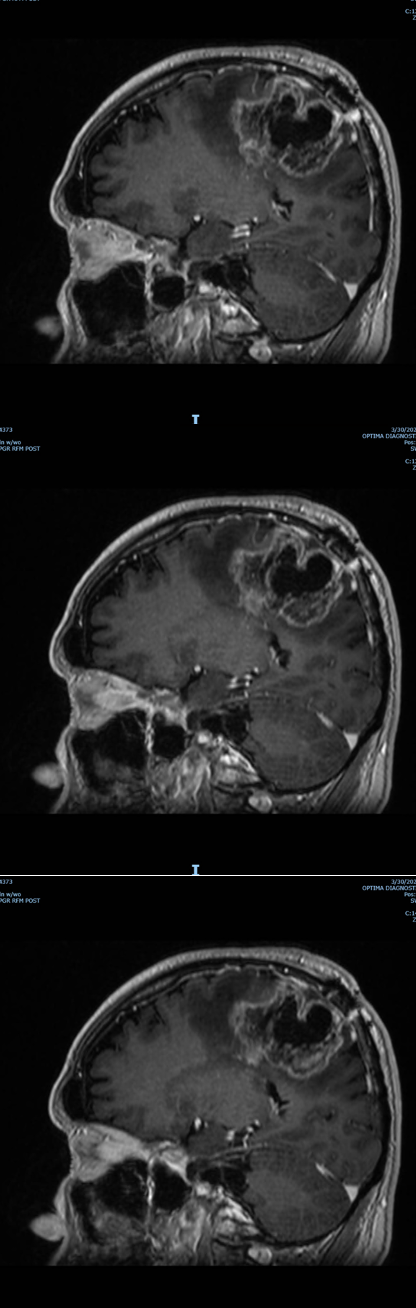

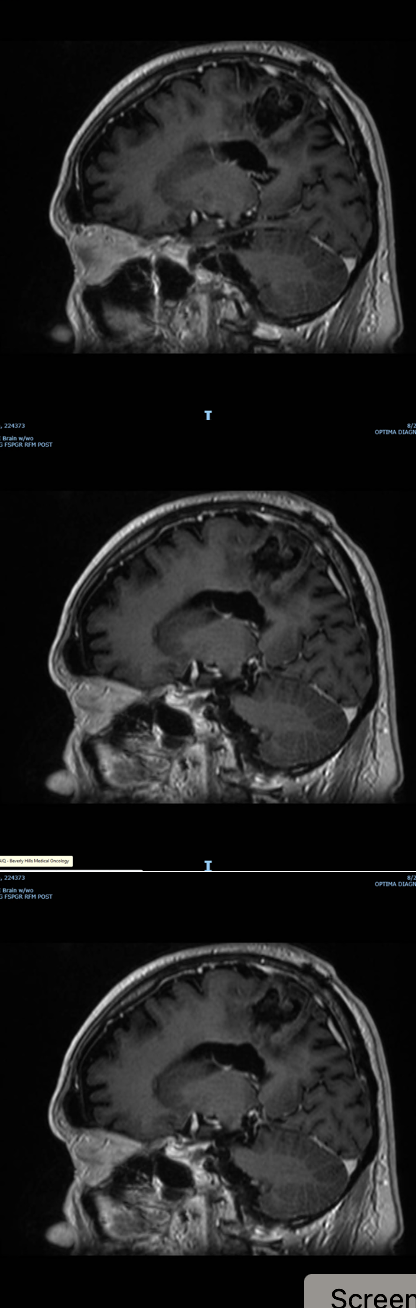

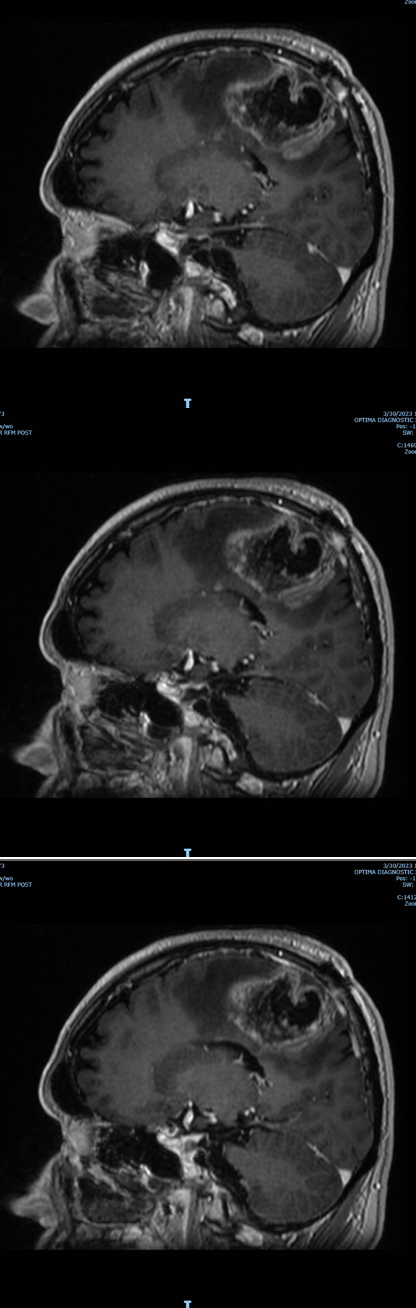

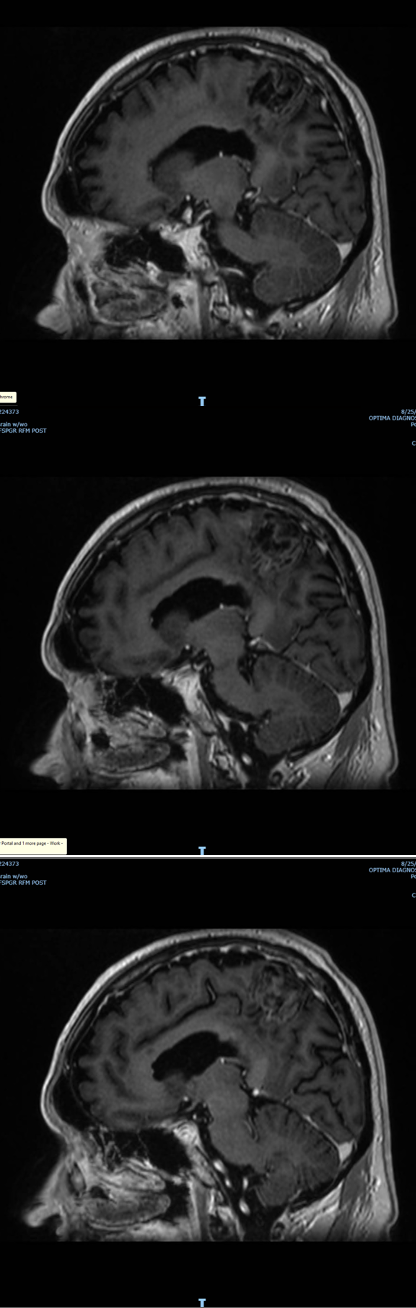

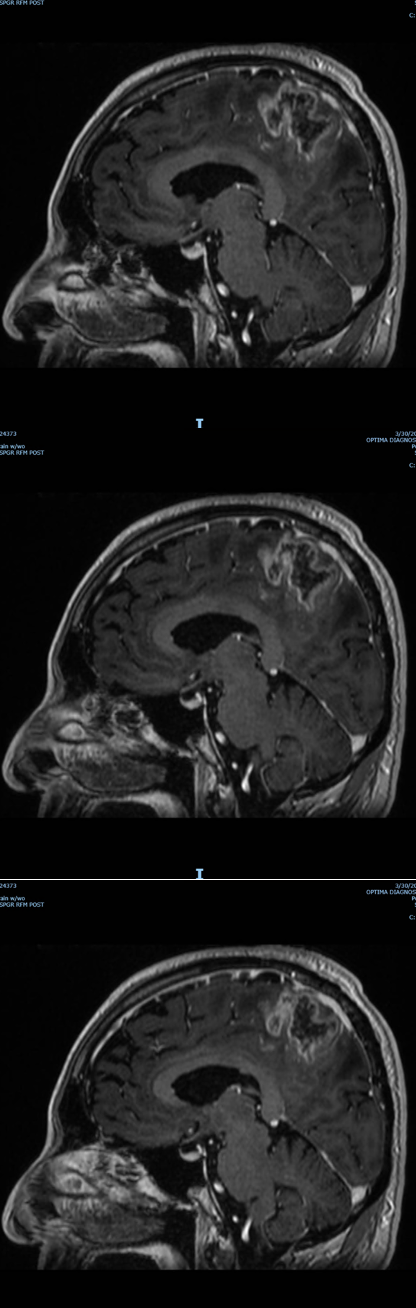

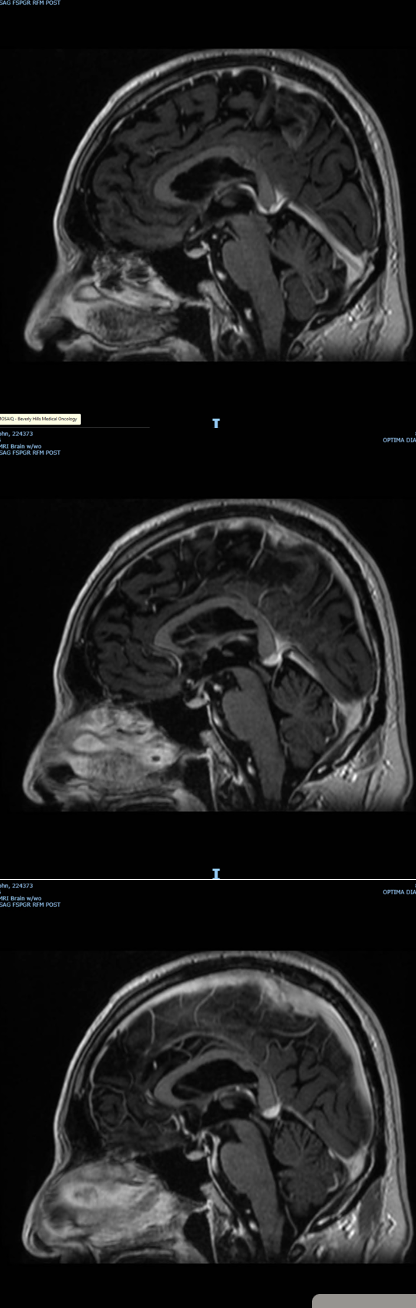

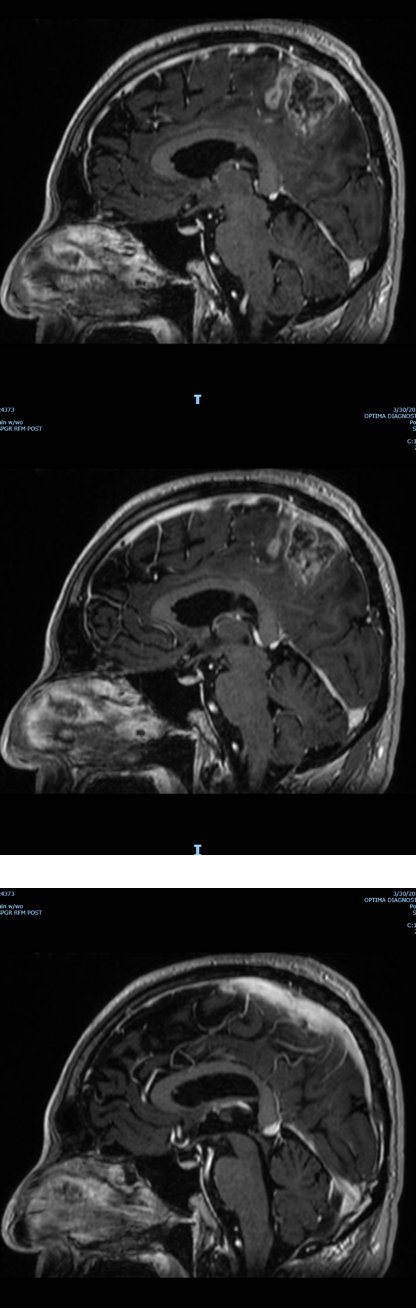

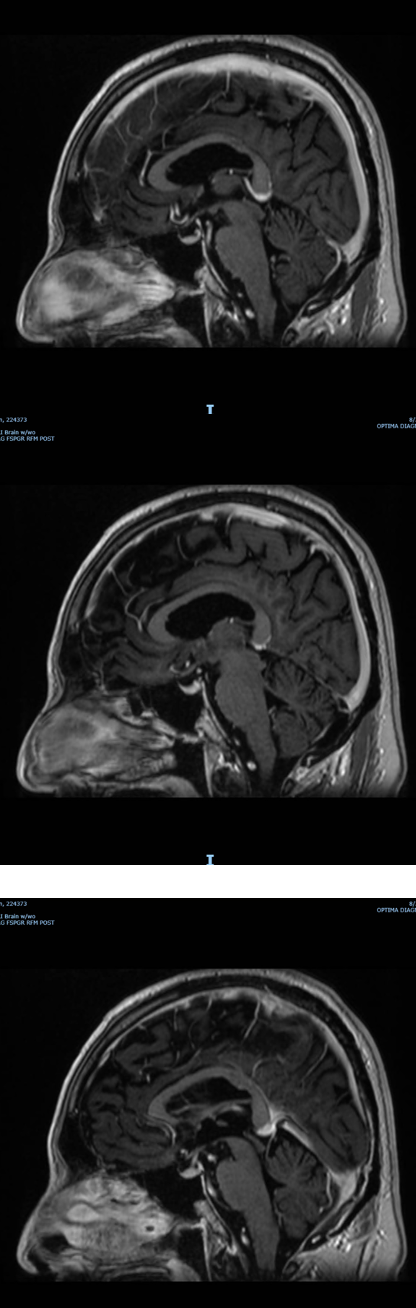

Supplement: vdad162_suppl_Supplementary_File [file vdad162_suppl_supplementary_file.docx]
